# Chicken caecal enterotypes in indigenous Kadaknath and commercial Cobb chicken lines are associated with *Campylobacter* abundance and influenced by farming practices

**DOI:** 10.3389/frmbi.2023.1301609

**Published:** 2023-12-04

**Authors:** Melanie C. Hay, Ankit T. Hinsu, Prakash G. Koringa, Ramesh J. Pandit, Po-Yu Liu, Mithil J. Parekh, Subhash J. Jakhesara, Xiaoxai Dai, Matteo Crotta, Bruno Fosso, Georgina Limon, Javier Guitian, Fiona M. Tomley, Dong Xia, Androniki Psifidi, Chaitanya G. Joshi, Damer P. Blake

**Affiliations:** ^1^ Department of Pathobiology and Population Sciences, Royal Veterinary College, Hatfield, United Kingdom; ^2^ Department of Veterinary Biotechnology, College of Veterinary Science and Animal Husbandry, Kamdhenu University, Anand, Gujarat, India; ^3^ Clinical Sciences and Services, Royal Veterinary College, Hatfield, United Kingdom; ^4^ School of Medicine, College of Medicine, National Sun Yat-sen University, Kaohsiung, Taiwan; ^5^ Department of Agricultural and Environmental Science, University of Milan, Milan, Italy; ^6^ Department of Biosciences, Biotechnologies and Environment, University of Bari “Aldo Moro”, Bari, Italy; ^7^ Veterinary Epidemiology, Economics and Public Health Group, World Organization for Animal Health (WOAH) Collaborating Centre for Risk Analysis and Modelling, The Royal Veterinary College, Hatfield, United Kingdom; ^8^ Gujarat Biotechnology Research Centre (GBRC), Department of Science and Technology, Government of Gujarat, Gandhinagar, Gujarat, India

**Keywords:** *Campylobacter*, chicken, enterotypes, microbiome, Random Forest

## Abstract

Identifying farming practices that decrease susceptibility to infectious diseases and optimise food conversion efficiency is valuable for chicken welfare and productivity, the environment, and public health. Enterotypes can be used to define microbial community phenotypes that have differential, potentially significant impacts on gut health. In this study, we delineated enterotypes by analysing the microbiomes of 300 indigenous Kadaknath and 300 commercial Cobb400 broiler chickens raised across 60 farms in western India. Using a compositional data approach, we identified three distinct enterotypes: PA1 (n=290), PA2 (n=142) and PA3 (n=67). PA1 and PA2 clustered more closely with each other than with PA3, however, PA2 had significantly lower alpha diversity than PA1. PA1 had a high Firmicutes: Bacteroides ratio, was dominated by *Faecalibacterium* and had a higher abundance of *Prevotellamassilia* than other enterotypes. PA2 was characterised by its low alpha diversity, a high abundance of the common taxa *Phascolarctobacterium* A and *Phocaeicola dorei* and a significantly higher *Campylobacter* abundance than PA1. PA3 had the highest Bacteroidota abundance of the three enterotypes and was defined by high prevalence of lower abundance taxa such as *CAG-831* and *Mucispirillum schaedleri*. Network analysis showed that all enterotypes have different proportions of competing Firmicutes-dominant and Bacteroidota-dominant guilds. Random Forest Modelling using defined farm characteristics was predictive for enterotype. Factors affecting enterotype include whether farms were open, enclosed or caged, the location of farms, whether visitors were allowed inside, the number of people in contact with the chickens, chicken line, the presence of dogs and whether flock thinning took place. This study suggests that enterotypes are influenced by farming practices, hence modification of practices could potentially be used to reduce the burden of zoonotic pathogens such as *Campylobacter*.

## 1 Introduction

Human populations continue to grow, especially in South Asia and Africa, with a consequent rise in demand for high-quality protein to meet nutritional needs (Michele, 2021). In response, intensification of chicken farming is increasing rapidly, but if this is not managed well then higher density production can pose direct threats to public health. Firstly, intensification increases the chances that potentially zoonotic agents, including avian influenza viruses, may jump from chickens to humans (Dhingra et al., 2018; Gilbert et al., 2021). Secondly, it can raise the likelihood that chicken meat, eggs or waste becomes contaminated by known human pathogens including *Campylobacter* species, *Salmonella enterica* serovars and enterotoxigenic strains of *Escherichia coli* (Berry and Wells, 2016). Thirdly, it increases selection of antimicrobial resistance (AMR) due to increased inappropriate prophylactic or therapeutic use of antibiotics (Rushton et al., 2014; Van Boeckel et al., 2015; Laxminarayan and Chaudhury, 2016; Mahalmani et al., 2019; Murray et al., 2022).

In this study we focus on the genus *Campylobacter*, the most common agents of food-borne illness globally (Kaakoush et al., 2015; EFSA Panel on Biological Hazards (BIOHAZ) et al., 2020) accounting for ~7% and ~10% of hospitalized adult and child diarrhoeal patients respectively in Kolkata, India (Mukherjee et al., 2013). Poultry are a frequently attributed source of *Campylobacter*; therefore, measures that reduce its carriage in chickens have beneficial impacts on public health; a 1000-fold decrease in caecal bacterial load results in a 58% reduction of the median relative risk of campylobacteriosis (EFSA Panel on Biological Hazards (BIOHAZ) et al., 2020). *Campylobacter* in chickens usually appears in the 3rd–4th week of life and is present in >90% of flocks by 7 weeks and up to 100% of flocks by 12 weeks (Evans and Sayers, 2000; Sakaridis et al., 2018; Sibanda et al., 2018). Although the introduction of *Campylobacter* into poultry houses is notoriously difficult to control, the relative abundance of *Campylobacter* within a flock can be reduced by farming practices and biosecurity measures such as stringent staff hygiene, physical barriers, reduced thinning, increased flock turnaround time and isolation from other livestock, domestic animals and rodents (Evans and Sayers, 2000; Ellis-Iversen et al., 2009; Sibanda et al., 2018; Alam et al., 2020; EFSA Panel on Biological Hazards (BIOHAZ) et al., 2020; Soro et al., 2020; Xu et al., 2021b). It has been hypothesised that such differences in abundance are due, at least in part, to impacts that farming practices have on the composition of the microbiome (Sakaridis et al., 2018; McKenna et al., 2020; Patuzzi et al., 2021; Wyszyńska and Godlewska, 2021).

The predominant sites for colonization of *Campylobacter* in broiler chickens are the paired caeca, host to the most abundant and diverse microbial communities of the chicken digestive tract with up to 10^10^ colony forming units (CFU) per gram of digesta (Rychlik, 2020). The importance of the caecal microbiome in maintaining chicken health and productivity is well recognised due to its role in food utilisation, and resistance to disease and colonisation by zoonotic pathogens (Clavijo and Flórez, 2018; Kers et al., 2018). An ideal “healthy” eubiotic caecal microbiome would be low in both human and chicken pathogens (lowering public health risk and maximising chicken welfare) and highly efficient at aiding food utilisation (improving food conversion and increasing meat production per carbon impact). Dysbiosis is the term applied to microbiomes when they become imbalanced and actively contribute to disease or suboptimal health phenotypes. This can be due to infection when high pathogen loads disrupt the normal balance, but imbalances can also occur without obvious infection causing shifts in the metabolism of the microbiome community and contributing to inefficient food usage and nutrient absorption (Ducatelle et al., 2018; Kogut, 2019; Aruwa et al., 2021). Identifying factors that contribute to “healthy” or dysbiotic microbiomes, and the underpinning mechanisms that are influenced by environmental or host genetic factors, are topics of great interest.

Many eubacterial taxa are highly abundant and prevalent in most chickens but found in different proportions to each other. Enterotyping is a method to classify microbial community phenotypes that have significant impacts on host health (Costea et al., 2018). Enterotypes may represent a state of gut bacteria in community equilibrium, with several factors including host genotype and environmental influences working to maintain a community within limits (Sommer et al., 2017). Distinct enterotypes can differ in their resilience to perturbations (and dysbiosis), affect susceptibility to disease or play a role in food conversion efficiency (Malard et al., 2021).

In this study, we investigated 300 indigenous Kadaknath and 300 commercial Cobb400 chickens from 60 farms in west India that followed a range of different farming practices. Our aims were firstly to distinguish distinct microbiome compositions (enterotypes) across a large population. Secondly, we investigated whether the enterotypes had differences in *Campylobacter* abundance. Finally, we looked at various farming practices to determine whether there were any factors that were associated with the occurrence of enterotypes.

## 2 Methods

### 2.1 Ethics approval and consent to participate

This study was carried out using welfare standards consistent with those established under the Animals (Scientific Procedures) Act 1986, an Act of Parliament of the United Kingdom. All protocols were approved by the Ethical Review Panel of Anand Agricultural University (AAU) (* now Kamdhenu University) and the Clinical Research Ethical Review Board (CRERB) of the Royal Veterinary College under the reference URN 2014 1280. Participating farmers were informed of the objectives of the study and written consent was obtained for the same.

### 2.2 Chicken lines and study design

**Figure 1:**
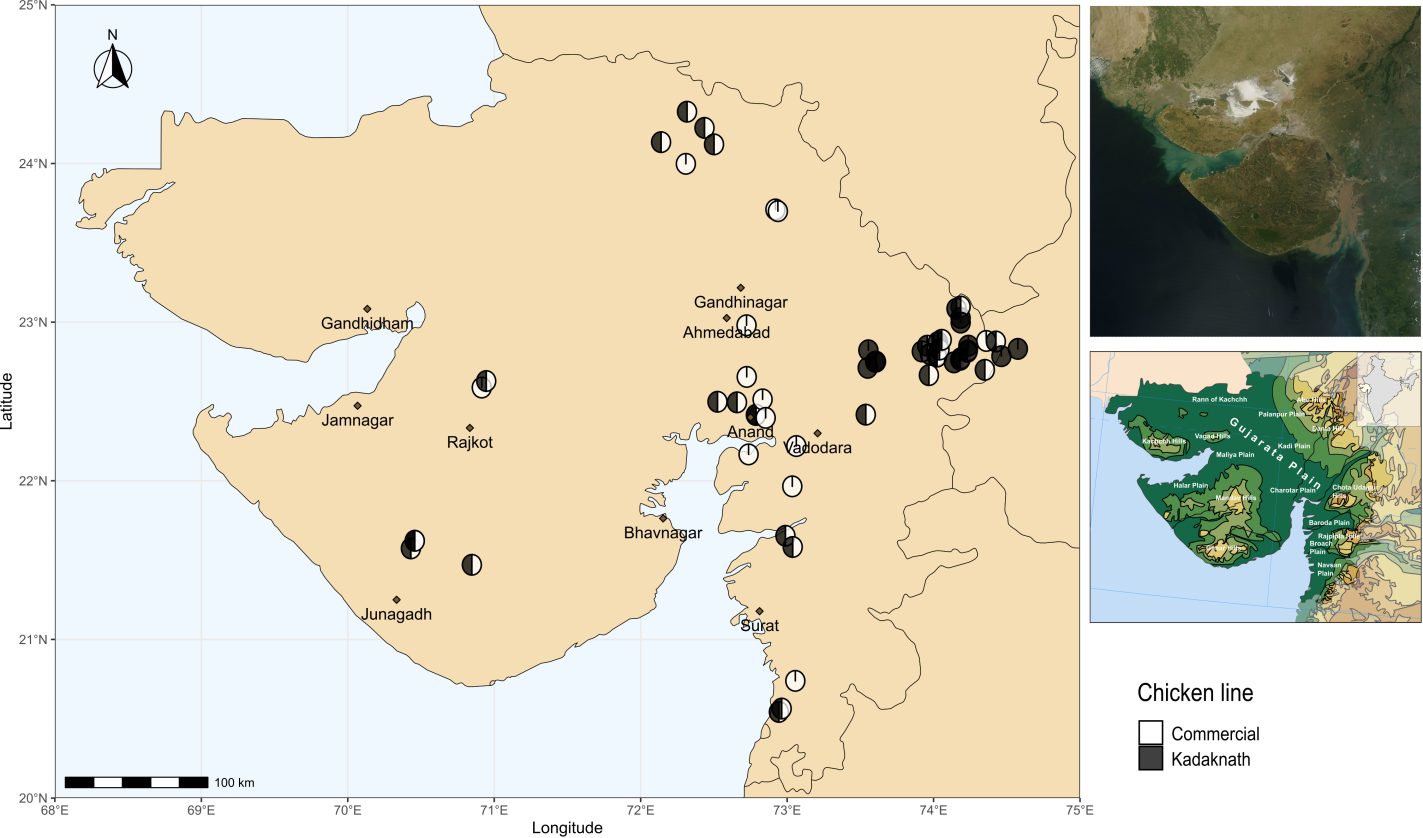
Map showing locations of sampling sites in western India. Cobb400-only farms are in white, Kadaknath-only farms are in black and Cobb400 + Kadaknath farms are in black and white. Top Right panel shows satellite imagery of Gujarat, and Bottom Right panel shows physical geography of Gujarat (Gujarat. (2023, October 26)). Source: Wikipedia, https://en.wikipedia.org/wiki/Gujarat (CC BY-SA 4.0 DEED).

Chicken lines and experimental design have been described previously (Hinsu et al., 2018). Briefly, Cobb400 broiler chickens are commercial meat-producing birds derived from Cobb500 and Cobb100 hybrids and widely used in India due to their good performance under tropical conditions (Dyck et al., 2004). Kadaknath chickens are an indigenous Indian line prized for their black meat and reputed to be resistant to some infectious diseases (Rout et al., 1992; Ramasamy et al., 2010). Sixty farms were included in the study, chosen to be convenient for field visits whilst covering a wide geographic range (**Figure 1**). Ten chickens were sampled from each farm to provide a total of 600 samples. Fifteen farms had only Cobb400 chickens, 15 farms had only Kadaknath, and 30 farms contained a mix of both chicken lines. From the mixed farms, five Cobb400 and five Kadaknath chickens were sampled. A standardised questionnaire was distributed to all farms, to collect farm characteristics on the same day as sampling (**Supplementary Data 1**).

### 2.3 Sample collection and DNA extraction

At each farm, ten chickens with no apparent signs of disease were selected, caught and euthanized by cervical dislocation. Both caecal pouches were opened immediately using sterile scissors and the contents scraped into sterile cryovials containing Bacterial Protect RNA reagent (QIAGEN, Germany) at an approximate 1:1 ratio (w/v). Samples were immediately stored in a portable freezer at −20°C, transported to the laboratory and stored at −80°C. Total DNA was extracted from the pooled caecal contents of each individual chicken using QIAamp Fast DNA Stool Mini kit (QIAGEN, Germany) following the manufacturer’s instructions with some modifications as described previously (Pandit et al., 2018). After extraction, DNA was treated with DNase free RNase (Macherey-Nagel, Germany) and the DNA concentration and quality assessed using a Qubit 2.0 fluorometer (Invitrogen, Thermo Fisher Scientific, MA, USA) and gel electrophoresis, respectively. Extracted DNA were stored at −20°C until further processing.

### 2.4 16S rRNA gene amplification and sequencing

The V3-V4 hypervariable region of eubacterial 16S rRNA gene was amplified using KAPA HiFi HotStart ReadyMix (Kapa Biosystems, UK) following the Illumina 16S rRNA amplicon library preparation protocol (Illumina, USA). All 600 samples were sequenced across nine Illumina MiSeq flow cells using an Illumina MiSeq desktop sequencer using 2 x 300bp PE sequencing. Samples were demultiplexed and adaptor sequences trimmed using Illumina analysis software V2.5 using default parameters. The 16S rRNA gene sequence data can be accessed on the Sequence Read Archive (SRA) ERP017060.

### 2.5 Bioinformatics

#### 2.5.1 Sequencing, quality control and taxonomic assignment using DADA2

Primer and adapter sequences were removed using cutadapt v 1.7.1 (Martin, 2011). All further processing and statistical analyses were performed in RStudio using R version 4.03. Reads were assigned to amplicon sequence variants (ASVs) using DADA2 (v. 1.18.0) (RRID : SCR_023519) (Callahan et al., 2016). The data were denoised individually by flow cell, then merged before chimera removal. Taxonomic assignment was performed to genus level using training fasta files derived from GTDB release 202 formatted for use with DADA2 (Alishum, 2021). The GTDB database was selected for taxonomic classification because it is based on whole genome phylogeny (Parks et al., 2018; Parks et al., 2020; Parks et al., 2022) and can be linked to whole genome relatives with functional information. Classification to species level was performed only when ASVs had 100% identity to the reference sequences.

#### 2.5.2 Data processing

The 16S rRNA gene abundance data were filtered and analysed using Phyloseq (V. 1.32.0) (RRID : SCR_013080) (McMurdie and Holmes, 2013). Analyses were performed on unrarefied datasets that had been filtered by relative abundance (RA, 0.1%, 0.01% or 0.001%) and prevalence (1%, 2%, 4%, 5% or 10%) and all combinations thereof (n=18; **Supplementary Data 2**). This was done to introduce slight variation into the data allowing us to identify samples that clustered together consistently (enterotype) and edge cases that fell between enterotypes. The first step of a compositional data approach is the transformation of data from counts to centre log ratio (clr) (Aitchison, 1982; Gloor et al., 2016; Gloor et al., 2017; Quinn et al., 2019). This transformation cannot be performed if there are any zeroes in the dataset, therefore zeroes were replaced with non-zero values using the cmultRepl() function in the R package zCompositions (Palarea-Albaladejo and Martín-Fernández, 2015), which imputes zeros based on a Bayesian-multiplicative replacement as recommended (Quinn et al., 2019). The clr transformation itself was performed using the R package robCompositions (Templ et al., 2011). Aitchison (AIT) distance (Euclidean distance of clr-transformed data) for the compositional approach was calculated using the package vegan (Oksanen et al., 2020).

#### 2.5.3 Clustering into enterotypes

Prior to clustering, ASVs counts were agglomerated to genus level. The ideal number of clusters was assessed using the maximum silhouette width, calculated using fviz_nbclust from the package factoextra (Kassambara and Mundt, 2020). Thereafter, samples were clustered using the partition around medoids (PAM) clustering algorithm available from the cluster R package (Maechler et al., 2022). Clustering was performed on the AIT distance matrix using each of the filtered datasets. The cluster membership of each sample within each of the filtered datasets was extracted and the intersections of cluster memberships used to assign enterotype. All steps were also performed using a traditional approach with clustering of Jensen Shannon Divergence (JSD) and Bray-Curtis (BC) distance and using different clustering methods (hcut and kmeans) for comparison (**Supplementary Data 3**).

#### 2.5.4 Visualising clusters using PhateR and Principal Component Analysis

Enterotype clusters were visualised with PhateR (Moon et al., 2019) using count data and the clr-transformed compositional datasets. Principal Component Analysis (PCA) on the clr-transformed data was done with FactoMineR (Lê et al., 2008; Husson et al., 2020) and visualised using factoextra (Kassambara and Mundt, 2020).

#### 2.5.5 Enterotype characteristics

For measures of alpha diversity, samples were rarefied to the smallest library size (11,111 reads), in all other cases, the dataset with the least filtering (RA=0.00001, prevalence =1%) was used for analysis which retained 99.8% of reads from the original dataset (**Supplementary Data 2**). Differential abundance of genera between enterotypes was measured using the R package ALDEx2 (RRID : SCR_003364) (Fernandes et al., 2014) and LEfSe analysis was conducted using an “all-against-one” approach (Segata et al., 2011). Co-occurrence network analysis for each enterotype was performed using the R package NetCoMi (Peschel et al., 2021). Single networks for each enterotype were constructed using sparCC (Friedman and Alm, 2012). Networks were also compared to each other to identify whether there were differences in connections between bacteria in the different enterotypes. The R code to generate all analyses and Figures are provided on GitHub.

#### 2.5.6 Random Forest Modelling

Random Forest Models (RFM) were run to determine whether farming characteristics can be used to determine enterotype (**Supplementary Data 4**). RFMs were constructed using the randomForest (V4.7-1.1) (Liaw and Wiener, 2002) and caret (V 6.0-92) (Kuhn, 2008) packages in R. There were two potential confounding factors considered when designing the RFMs. Firstly, the likelihood of sharing an enterotype is much higher for chickens from the same farm than for chickens on different farms. Secondly, the number of chickens with each enterotype differs, so training data needed to be balanced to ensure the model could discern equally between groups. To account for these potential confounders, the training and testing datasets were partitioned 80:20 prior to any processing, whilst ensuring that chickens from the same farm could not appear in both splits to prevent “data leakage” inflating the accuracy of the models (Kapoor and Narayanan, 2022). In addition, while training the models we used groupKFold to split data in the training and validation datasets such that samples from a single farm could not appear in both. Furthermore, we used trainControl to perform 10x repeated cross-validation repeated seven times when training the models and used down sampling to balance the enterotype classes. Finally, the entire process from data partition to confusion matrix was repeated on five different test:train splits. This was particularly useful to see whether models based on different data splits still ranked important variables for each enterotype consistently. Plots were generated using the package ggplot2 (V 3.3.6) (Wickham, 2016).

## 3 Results

### 3.1 The chicken caecal microbiome is highly diverse

After quality control and processing through DADA2 there were 4,658,348 reads assigned to 20,888 unique ASVs. Library size ranged from 11,111 reads to 266,317 reads, with a mean of 77,646 reads. GTDB assigned ASVs to 34 Phyla, 55 Classes, 124 Orders, 256 Families and 633 Genera. Five samples were removed as outliers based upon low Shannon Diversity (> 1.5* interquartile range (IQR)) (**Supplementary Data 5**), leaving 595 samples containing 625 genera.

Across all samples, the most abundant phyla were the Firmicutes (specifically Firmicutes_A, followed by Firmicutes and Firmicutes_C). The Bacteroidota were the second most abundant, and Campylobacterota were third most abundant, followed by the Proteobacteria. Cyanobacteria, Verrucomicrobia, Synergistota and Fusobacterota phyla were also frequently present at lower abundance. At the Genus level, the most abundant genera were *Phocaeicola*, *Phascolarctobacterium*, *Faecalibacterium*, *Mediterraneibacter*.

### 3.2 Characteristics of enterotypes

**Figure 2:**
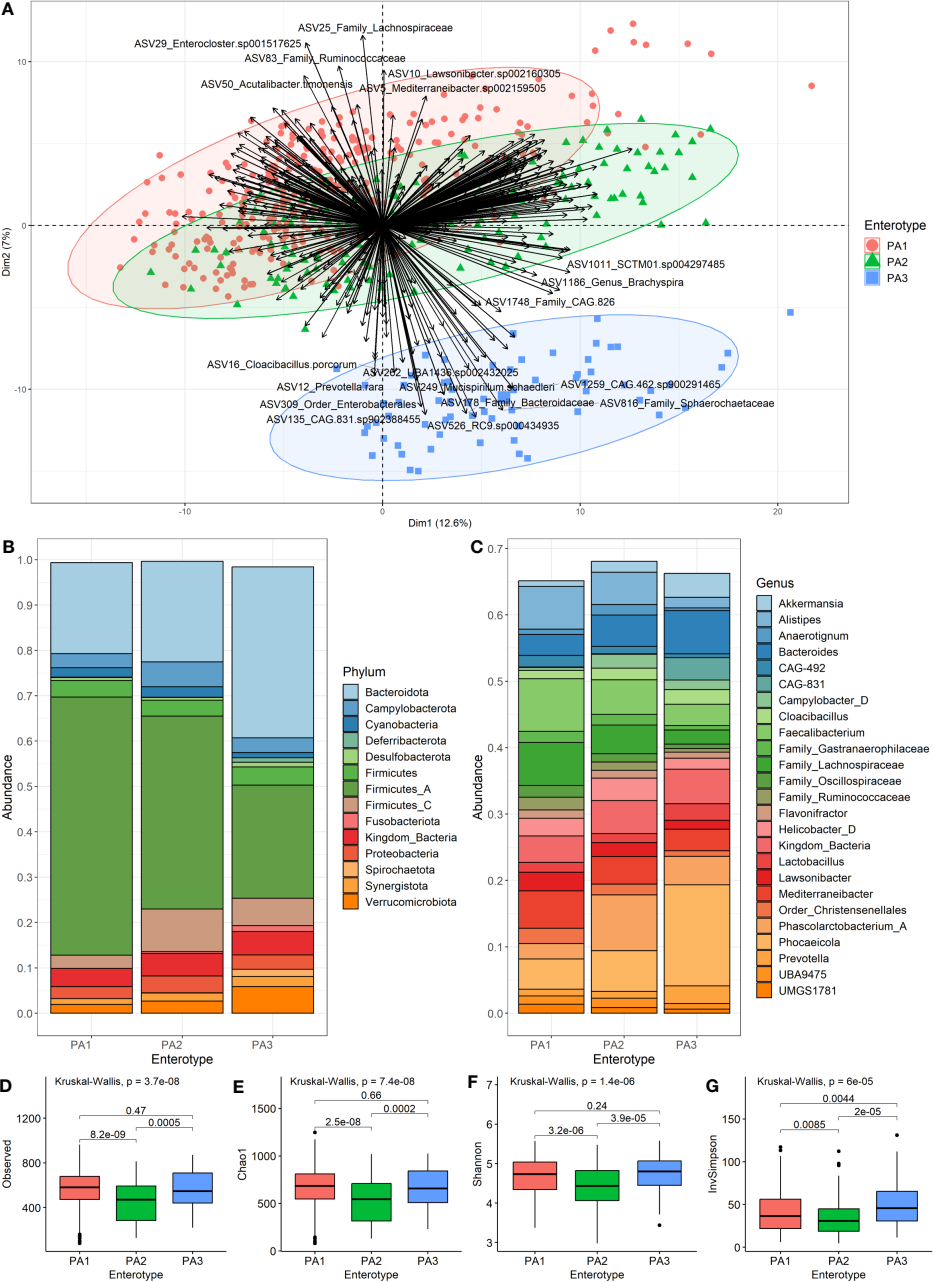
Characteristics of the three enterotypes. **(A)** is a PCA Biplot showing clusters based on the PAM clustering of samples by Aitchison distance, as well as the genera contributing to the spread of the samples. **(B)** shows the community composition (mean relative abundance) of the three enterotypes at the phylum level. **(C)** shows the community composition (mean relative abundance) of the enterotypes at the genus level. **(D–G)** show alpha diversity of enterotype groups. **(D)** Species-richness, **(E)** Chao1, **(F)** Shannon Index and **(G)** Inverse Simpson. Pairwise comparisons using Wilcoxon rank sum test with BH (Benjamini-Hochberg) correction for multiple testing were used to detect significant differences between the group means.

The ideal number of clusters suggested for PAM clustering of compositional data (AIT distance) was k=2 clusters (**Supplementary Data 6**). Since enterotypes are “areas of high density” in a multidimensional space, we sought to identify these “density peaks” and eliminate intermediate samples by taking the intersection of PAM clusters, i.e., samples that clustered together across all 18 datasets. However, when the resultant datasets were generated, it was apparent that three enterotypes could be described. These were numbered according to how common they were as PA1 (n=290), PA2 (n=142) and PA3 (n=67) for PAM-clusters based on Aitchison distance. Hence, although we used k=2, we ended up with three groupings and this was because different filtering thresholds resulted in a split of the largest group (**Supplementary Data 7**). PA3 was identified as separate from (PA1+PA2) most of the time, so PA1 and PA2 can be viewed as subsets of a larger group, whilst PA3 is a more robust separate cluster (**Figure 2A**). Of interest, PAM clustering of AIT versus the BC or JSD distance data differed significantly (**Supplementary Data 8**). Other versions of the data are available for different clustering methods, different values of k and different distance measures, and are named according to this same convention (e.g., PJ2, PJ8, PB2, PB8, PJBA).

We merged the samples of each enterotype to compare the mean community composition between enterotypes at phylum (**Figure 2B**) and genus (**Figure 2C**) levels (**Supplementary Data 9**). The most striking different between enterotypes was the ratio of Firmicutes to Bacteroides (F:B). PA1 had the highest F:B ratio (3.16), followed by PA2 (2.50) and PA3 (0.93). The Campylobacterota phylum was highest in PA2 (5.47%), and PA3 had many members of the Spirochaetota phylum (1.5%) which was almost absent in the other enterotypes. The most abundant genera were *Faecalibacterium* (7.92%), *Phascolarctobacterium A* (8.39%) and *Phocaeicola dorei* (15.21%) for enterotypes PA1, PA2 and PA3, respectively. PA3 had quite a large abundance of CAG-831 (3.36%), a distinct species, but close relative of *Alistipes*, which was not present in the other enterotypes.

There were statistically significant differences between PA1 and PA2 and between PA2 and PA3 for all measures of alpha diversity (**Figures 2D–G**). There was no significant difference detected between PA1 and PA3 for species-richness (**Figure 2D**), Chao1 (**Figure 2E**) or Shannon index (**Figure 2F**), however there was a significant difference for Inverse Simpson (**Figure 2G**). The PA2 enterotype had significantly lower alpha diversity than both other enterotypes for all measures (**Figures 2D–G**).

### 3.3 Differentially abundant genera between enterotypes

**Figure 3:**
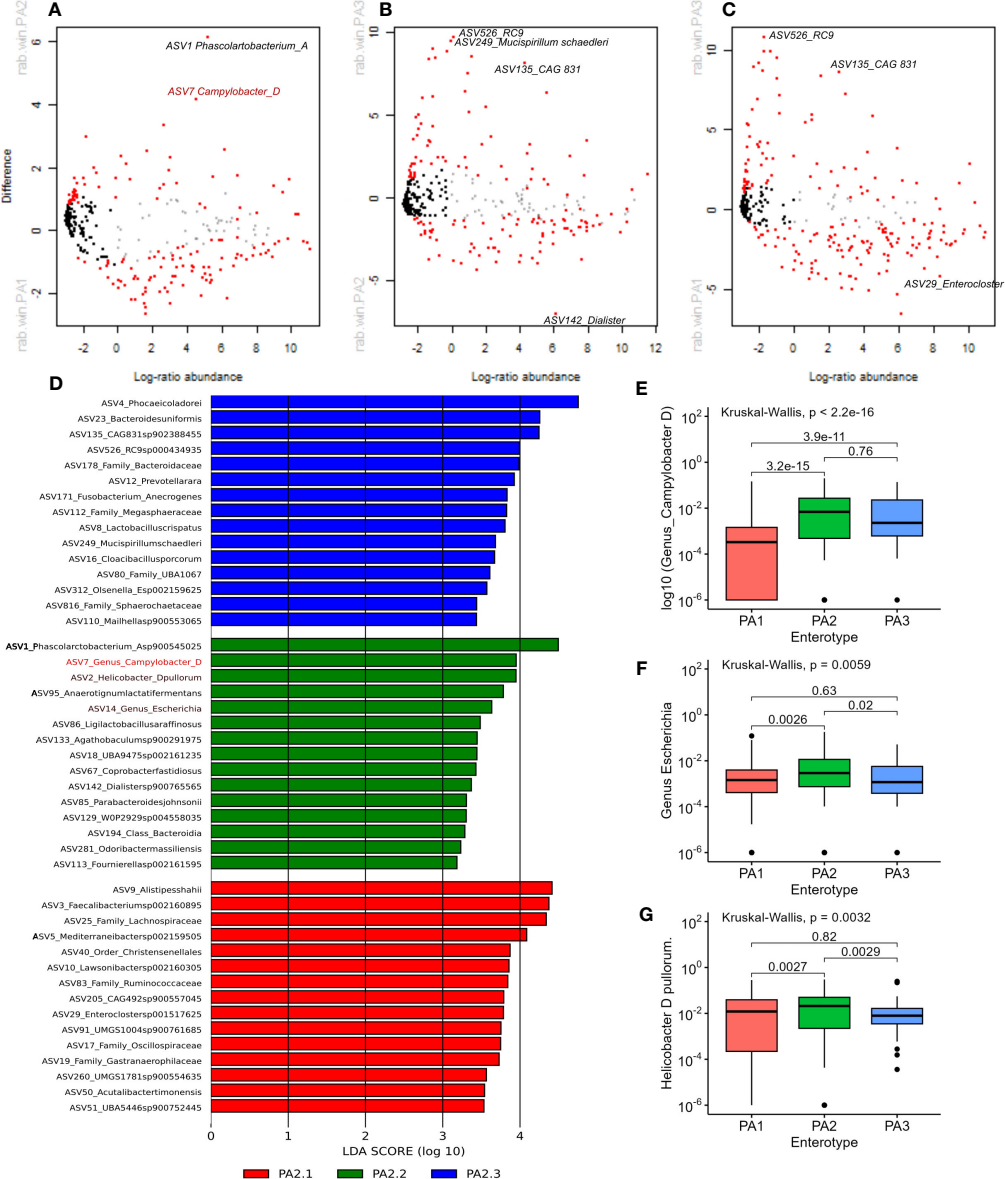
**(A–C)** Aldex2 plot shows differentially abundant genera between enterotype groups. **(A)** PA1 vs PA2, **(B)** PA1 vs PA3, **(C)** PA2 vs PA3. In all plots, red represents differentially abundant features (q < 0.1) using both Welchs’ and Wilcoxon tests, blue represents differentially abundant features (q < 0.1) detected by either Welchs’ or Wilcoxon tests, grey represents abundant but non-differentially abundant genera and black represent rare non-differentially abundant genera. **(D)** shows the top 15 discriminant genera in each Enterotype as identified by LEfSe analysis using “all against 1” approach. **(E, F)** Comparison of relative abundance of potential pathogens in chicken enterotypes. The mean RA of **(E)** *Campylobacter D*, **(F)** *Escherichia*, and **(G)** *Helicobacter D pullurom* are compared across enterotypes.

Methods to analyse microbiome differential abundance produce differing results (Nearing et al., 2022), therefore we used two different methods to detect differentially abundant genera. ALDEx2 is a differential abundance test that considers the compositional nature of data to look for significant differences in abundance (Fernandes et al., 2014) and has shown consistency across different datasets (Nearing et al., 2022). LEfSe analysis is commonly used in microbiome analysis (Segata et al., 2011) and identified many of the same genera highlighted by ALDEx2. Differentially abundant genera between enterotypes are shown in **Figure 3**.

PA1 and PA2 were more like each other than either was to PA3, which is evident from the higher overlap, lower BH corrected Welch test p-values and lower effect sizes (**Supplementary Data 10**). Nevertheless, 88 out of 371 genera had significant adjusted p-values < 0.01. The four most significant discriminant genera between PA1 and PA2 were, *Phascolarctobacterium A* (PA1:RA = 2.33%, PA2:RA = 8.39%; p=3.97E-17), *Phocaeicola dorei* (PA1:RA = 4.57%, PA2:RA = 6.14%, p*=*1.84E-12) *Prevotellamassilia* (PA1: RA 0.53%, p=9.93E-11) and *Campylobacter* (PA1: 0.43%, PA2: 2.1% p=4.67E-10).

Between PA1 and PA3, 164 out of 380 genera were significantly differentially abundant (P < 0.01). There were six genera strikingly more abundant in PA3 than PA1 (**Supplementary Data 10**). These were CAG 831 (p=1.38E-94), *Mucispirillum schaedleri* (p=8.75E-64), *Cloacibacillus porcorum* (p=1.52E-51), *Duodenibacillus* (p=3.89E-35), *Mailhella* (p=7.72E-34), and *Phocaeicola dorei* (p=2.05E-32). Between PA2 and PA3, 106 of 375 genera were significantly differentially abundant. The most discriminant genera were *Mucispirillum schaedleri* (p=3.96E-52), CAG.831 (p=1.59E-46), an uncharacterised genus from order Enterobacterales (p=2.43E-26), RC9 (p=2.32E-25) and an uncharacterised genus from family *Sphaerochaetaceae* (p=2.69E-24). The most significant differences between PA1 vs PA3 and PA2 vs PA3 were due to genera that were significantly more abundant in PA3. The top discriminant genus that was significantly more abundant in PA1 and PA2 than PA3 was the same uncharacterised genus belonging to family *Lachnospiraceae* (p=6.57E-31 and p=1.07E-21 respectively).

**Figure 4:**
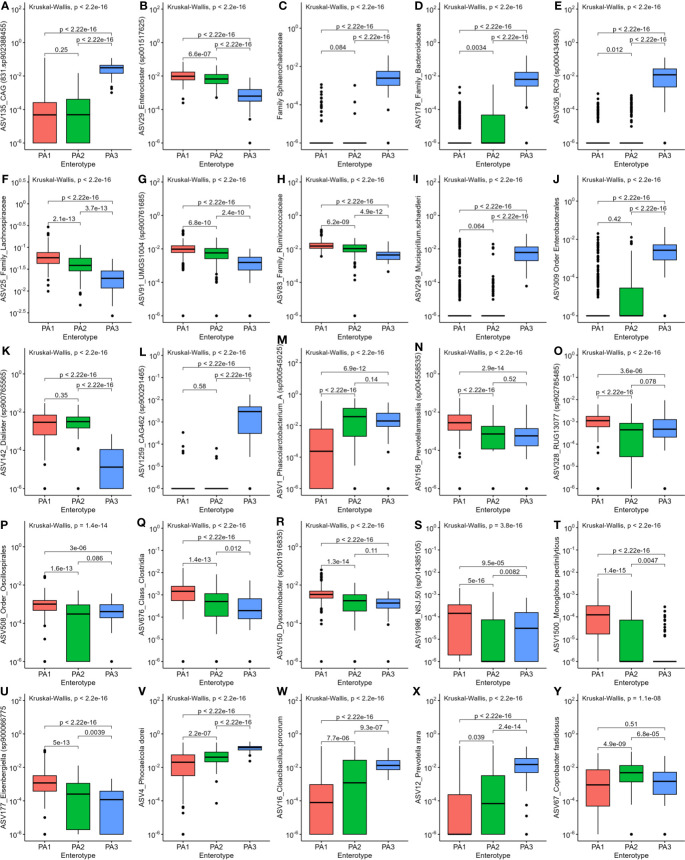
Comparison of relative abundance of the most differentially abundant genera between the three enterotypes. **(A)** CAG831, **(B)** *Enterocloster*, **(C)** *Sphaerochaetaceae*, **(D)** Family_Bacteroidaceae, **(E)** ASV526_RC9, **(F)** Lachnospiraceae, **(G)** ASV91_UMGS, **(H)** *Ruminococcaceae*, **(I)** *Mucispirillum*, **(J)** *Enterobacterales*, **(K)** *Dialister*, **(L)** CAG462, **(M**) *Phascolarctobacterium*, **(N**) *Prevotellamassilia*, **(O)** RUG13077, **(P)** Oscillospirales, **(Q)** Clostridia, **(R)** *Dysosmobacter*, **(S)** NSJ.50, **(T**) *Monoglobus*, **(U)** *Eisenbergiella*, **(V)** *Phocaeicola*, **(W**) *Cloacibacillus*, **(X)** *Prevotella*, **(Y)** *Coprobacter*.

LEfSe analysis indicated that potential human pathogens viz Campylobacter_D (including *Campylobacter coli* and *Campylobacter jejuni*, the cause of human campylobacteriosis) (**Figure 3D, E**), *Escherichia* (**Figure 3D, F**) (including enterotoxigenic strains and also a reservoir of AMR genes) and *Helicobacter* species (**Figures 3D–G**) were differentially abundant in enterotype PA2. Although *Campylobacter_D* was highly prevalent across all samples (445 samples) and enterotypes (PA1, n=215/290, PA2, n=102/142, PA3, n=55/67), the relative abundance was significantly higher in PA2 and PA3 than in PA1 (**Figure 3E**), whereas the genus *Escherichia* was slightly more abundant in PA2 than PA1 or PA3 (**Figure 3F**). Of interest, the dominant *Helicobacter* species differed between enterotypes. *Helicobacter pullurom* was more abundant in PA1 and PA2 than PA3 (**Figure 3G**), whereas a different *Helicobacter*_*F* clade was found in PA3, and completely absent from PA1 and PA2. The functional significance of the enterotype preference for these different *Helicobacter* species is worth investigating further. **Figure 4** shows boxplots of the relative abundance of the genera detected as most differentially abundant by ALDEeX2.

### 3.4 Understanding the enterotype “community” via co-occurrence networks

To understand how external factors influence enterotype, it is useful to examine bacterial interactions within the enterotypes. Bacterial genera with strong positive correlations are likely in symbiosis with each other or influenced by the same environmental drivers and thus may be important determinants for the phenotype. We refer to clusters of co-occurring and positively correlated bacteria as “ecological guilds” in line with the established definition of guilds in macro-ecological studies (Simberloff and Dayan, 1991) and recent microbiome research (Wu et al., 2021; Frioux et al., 2023).

**Figure 5:**
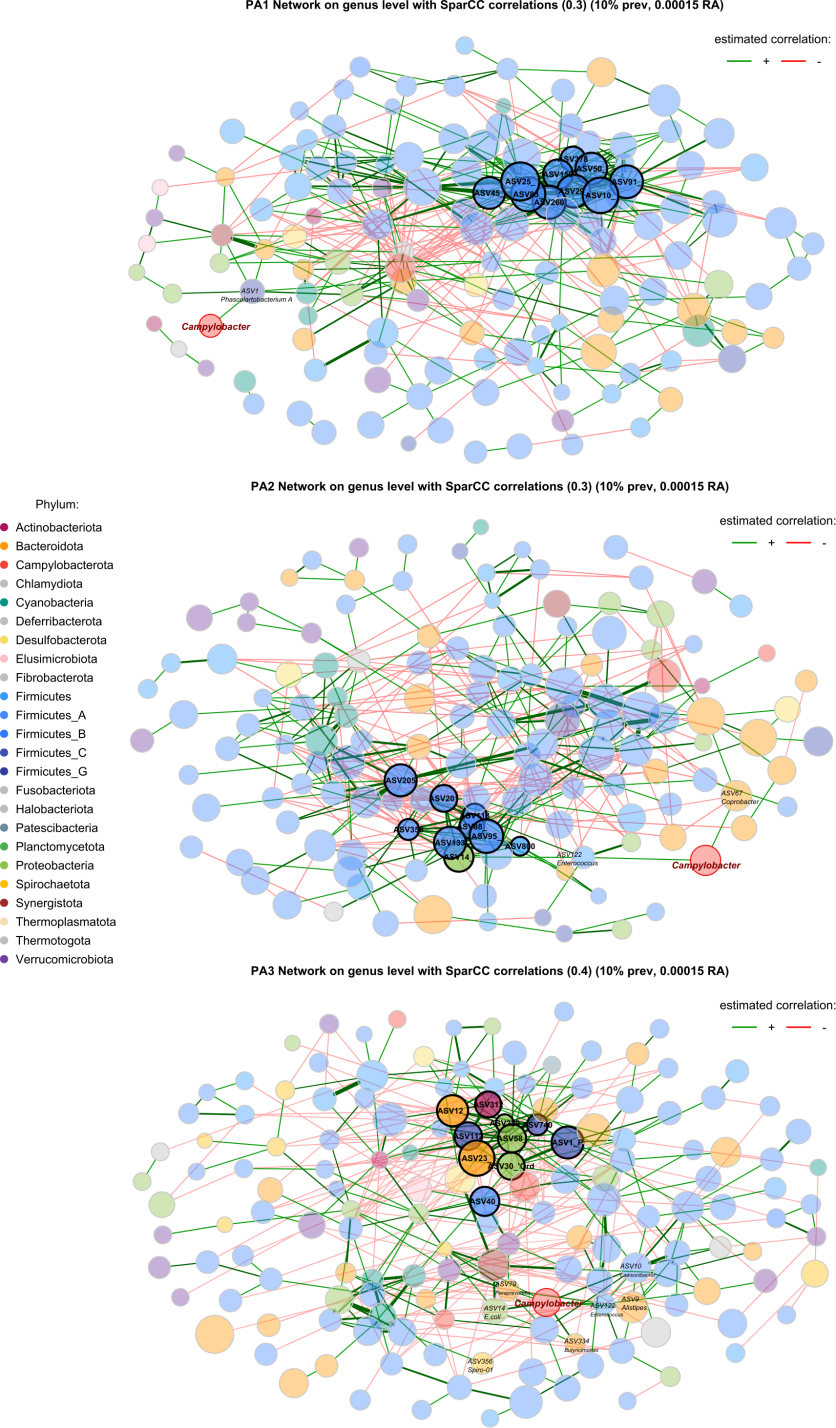
Co-occurrence network based on SparCC correlations between genera in each enterotype. Node size represents the clr (abundance) of the genus, node colour indicates phylum, edges represent correlations, and the width of the edge represents correlation strength. Nodes with a highlighted border are hub nodes (**Table 1**). The community connectivity increases from PA1 to PA2 to PA3, with PA3 showing a particularly connected community. The *Campylobacter* node is highlighted with a red border. Red edges represent negative correlations and green edges represent positive correlations.

A striking characteristic of the co-occurrence networks was the appearance of two guilds within the chicken caecal microbiome. Enterotypes PA1 and PA2 had a Firmicutes-dominant guild, and PA3 had a Bacteroidota- and Proteobacteria-dominant guild (**Table 1**, **Figure 5**, **Supplementary Data 11**). Hub nodes represent genera that have a greater influence on structuring the network. In PA1 all hub nodes are in a Firmicutes-dominant guild, whereas in PA3 they are all in the Bacteroides and Proteobacteria‐dominant guild, with PA2 having hub nodes from the Firmicutes and Proteobacteria.

**Table 1:** Genera that form network hubs in each of the two guilds.

	Guild 1 (Firmicutes-dominant)	Guild 2 (Bacteroides + Proteobacteria-dominant)
**PA1**	ASV10_Lawsonibacter sp002160305 (RS_GCF_002160305_1) ASV25_Family_Lachnospiraceae ASV29_Enterocloster sp001517625 (RS_GCF_001517625_2) ASV45_Anaerosacchariphilus sp002160825 (RS_GCF_002160825_1) ASV50_Acutalibacter timonensis (RS_GCF_900048895_1) ASV83_Family_Ruminococcaceae ASV91_UMGS1004 sp900761685 (RS_GCF_900761685_1) ASV150_Dysosmobacter sp001916835 (RS_GCF_014297285_1 ASV260_UMGS1781 sp900554635 (GB_GCA_900554635_1) ASV378_Caprobacter sp004103755 (RS_GCF_004103755_1)	
**PA2**	ASV14_Genus_Escherichia ASV88_UMGS1872 sp014237695 (GB_GCA_014237695_1) ASV95_Anaerotignum lactatifermentans (RS_GCF_900142265_1) ASV113_Fournierella sp002161595(RS_GCF_002161595_1) ASV133_Agathobaculum sp900291975(RS_GCF_900291975_1) ASV201_Gemmiger variabilis(RS_GCF_000157955_1) ASV205_CAG-492 sp900557045(GB_GCA_900557045_1) ASV358_Lachnoclostridium_A edouardi(RS_GCF_900240245_1) ASV800_Massilicoli timonensis(RS_GCF_900199515_1)	
**PA3**		ASV1_Phascolarctobacterium_A sp900545025 (GB_GCA_900545025_1) ASV12_Prevotella rara (RS_GCF_001275135_1 ASV23_Bacteroides uniformis (RS_GCF_000154205_1) ASV40_Order_Christensenellales ASV58_CAG-521 sp000437635 (GB_GCA_000437635_1) ASV112_Family_Megasphaeraceae ASV309_Order_Enterobacterales ASV312_Olsenella_E sp002159625 (RS_GCF_002159625_1) ASV740_Genus_Anaeroglobus ASV2291_Class_Gammaproteobacteria

In PA1, *Campylobacter* is positively correlated with *Phascolartobacterium_A*, however the relative abundance of both genera is significantly lower in PA1 than in other enterotypes, and *Phascolartobacterium_A* is negatively correlated with the highly abundant Firmicutes-dominant guild. In PA2, *Campylobacter* is positively associated with *Coprobacter* and *Enterococcus. Enterococcus* is in turn positively correlated with *E.coli*, which is also a hub genus in PA2 and significantly differentially abundant. In PA3, there were many more significant associations between bacteria throughout the network, and many more associations with *Campylobacter* specifically. Like PA2, *Campylobacter* was positively associated with *E.coli* in PA3 and had a direct positive association with *Enterococcus.*

### 3.5 Association of environmental factors with enterotype

Using Network analysis, we consistently saw caecal microbiomes from chickens from the same farm clustering together, particularly when analysing at ASV level. This suggests that environmental factors contribute to determining enterotype and by clustering chickens with similar enterotypes from several farms, it was possible to explore whether these farms had environmental factors in common that might contribute to similarities in microbiome. We created five different RFMs, based on different combinations of farms in the train and test splits (80:20) (**Supplementary Data 12** **).** The RFMs were trained on between 409 and 419 samples and used 28 predictors to classify samples into three enterotype classes (**Supplementary Data 4**). The performance of all five models was tested against the test data (consisting of 80 to 90 samples), and a confusion matrix generated as well as overall statistics and statistics by class (**Table 2**). The majority class PA1 made up 54–67% of the test data in the different models, which means that accuracy needed to exceed 54–67% to be better than the No Information Rate (NIR). All 5 models did better than the NIR. For RFM-1 and RFM-5 improvement over the NIR was statistically significant (RFM-1: p=0.0275, RFM-5: p=0.0001) and for the remaining three models, the improvement approached significance (RFM2: p=0.0998, RFM-3: p=0.0507, RFM-4: p=0.0690). The sensitivity and specificity of each model differed for each enterotype. PA3 (n=67) was the most discrete enterotype, however it was also the least common, and occurred in only a few (12) farms. Because of this rarity, and because we split by farm to prevent data leakage, very few farms could be used to train and test PA3 hence the models had either 100% (4/5 models) or 0% (1/5) sensitivity. The sensitivity and specificity of PA1 and PA2 varied by model, and the classifier was more likely to mistake these two classes, which is perhaps unsurprising as these two enterotypes were more like each other than PA3, and both were often represented on the same farm.

**Table 2:** Table comparing the performance of five Random Forest Models based on different splits of the data.

	RF1				RF2			RF3				RF4			RF5		
Confusion Matrix																	
	Reference				Reference			Reference				Reference			Reference		
Prediction	**PA1**	**PA2**		**PA3**	**PA1**	**PA2**	**PA3**	**PA1**	**PA2**		**PA3**	**PA1**	**PA2**	**PA3**	**PA1**	**PA2**	**PA3**
**PA1**	**27**	8		0	**45**	0	10	**44**	11		0	**39**	10	0	**50**	10	0
**PA2**	11	**17**		0	10	**17**	1	5	**16**		0	13	**11**	0	1	**13**	0
**PA3**	5	4		**8**	1	0	**0**	6	5		**3**	1	0	**10**	1	0	**5**
Statistics by Class																	
	**PA1**	**PA2**		**PA3**	**PA1**	**PA2**	**PA3**	**PA1**	**PA2**		**PA3**	**PA1**	**PA2**	**PA3**	**PA1**	**PA2**	**PA3**
Sensitivity	0.628	0.586		1.000	0.804	1.000	0.000	0.800	0.500		1.000	0.736	0.524	1.000	0.962	0.565	1.000
Specificity	0.784	0.784		0.875	0.643	0.836	0.986	0.686	0.914		0.874	0.677	0.794	0.987	0.643	0.983	0.987
Pos Pred Value	0.771	0.607		0.471	0.818	0.607	0.000	0.800	0.762		0.214	0.796	0.458	0.909	0.833	0.929	0.833
Neg Pred Value	0.644	0.769		1.000	0.621	1.000	0.868	0.686	0.768		1.000	0.600	0.833	1.000	0.900	0.849	1.000
Prevalence	0.538	0.363		0.100	0.667	0.202	0.131	0.611	0.356		0.033	0.631	0.250	0.119	0.650	0.288	0.063
Detection Rate	0.338	0.213		0.100	0.536	0.202	0.000	0.489	0.178		0.033	0.464	0.131	0.119	0.625	0.163	0.063
Detection Prevalence	0.438	0.350		0.213	0.655	0.333	0.012	0.611	0.233		0.156	0.583	0.286	0.131	0.750	0.175	0.075
Balanced Accuracy	0.706	0.685		0.938	0.723	0.918	0.493	0.743	0.707		0.937	0.707	0.659	0.993	0.802	0.774	0.993
Overall Statistics																	
Accuracy	0.650				0.738			0.700				0.714			0.850		
95% CI	(0.5352, 0.7533)				(0.6307, 0.828)			(0.5943, 0.7921)				(0.6053, 0.8076)			(0.7526, 0.92)		
No Information Rate	0.538				0.667			0.611				0.631			0.650		
P-Value [Acc > NIR]	**0.0275**				0.0998			0.0507				0.0690			**0.0001**		
Kappa	0.433				0.470			0.443				0.476			0.672		
	RF1							RF3							RF5		
Confusion Matrix																	
	Reference							Reference							Reference		
Prediction	**PA1**		**PA2**		**PA3**			**PA1**		**PA2**		**PA3**			**PA1**	**PA2**	**PA3**
**PA1**	**21**		8		0			**40**		8		0			**46**	14	0
**PA2**	21		**21**		0			9		**19**		0			5	**9**	0
**PA3**	1		0		**8**			6		5		**3**			1	0	**5**
Statistics by Class																	
	**PA1**		**PA2**		**PA3**			**PA1**		**PA2**		**PA3**			**PA1**	**PA2**	**PA3**
Sensitivity	0.4884		0.7241		1			0.7273		0.5938		1			0.8846	0.3913	1
Specificity	0.7838		0.5882		0.9861			0.7714		0.8448		0.87356			0.5	0.9123	0.9867
Pos Pred Value	0.7241		0.5		0.8889			0.8333		0.6786		0.21429			0.7667	0.6429	0.8333
Neg Pred Value	0.5686		0.7895		1			0.6429		0.7903		1			0.7	0.7879	1
Prevalence	0.5375		0.3625		0.1			0.6111		0.3556		0.03333			0.65	0.2875	0.0625
Detection Rate	0.2625		0.2625		0.1			0.4444		0.2111		0.03333			0.575	0.1125	0.0625
Detection Prevalence	0.3625		0.525		0.1125			0.5333		0.3111		0.15556			0.75	0.175	0.075
Balanced Accuracy	0.6361		0.6562		0.9931			0.7494		0.7193		0.93678			0.6923	0.6518	0.9933
Overall Statistics																	
**Accuracy**	0.625							0.6889							0.75		
**95% CI**	(0.5096, 0.7308)							(0.5826, 0.7823)							(0.6406, 0.8401)		
**No Information Rate**	0.5375							0.6111							0.65		
**P-Value [Acc > NIR]**	0.0718							0.07853							0.03683		
**Kappa**	0.3787							0.4427							0.4536		

Random Forests (RF) 1–5 were first run with alpha diversity to distinguish between chickens on a single farm. RFs were there run on the same farm splits as RF1, RF3 and RF5 without alpha diversity.

**Figure 6:**
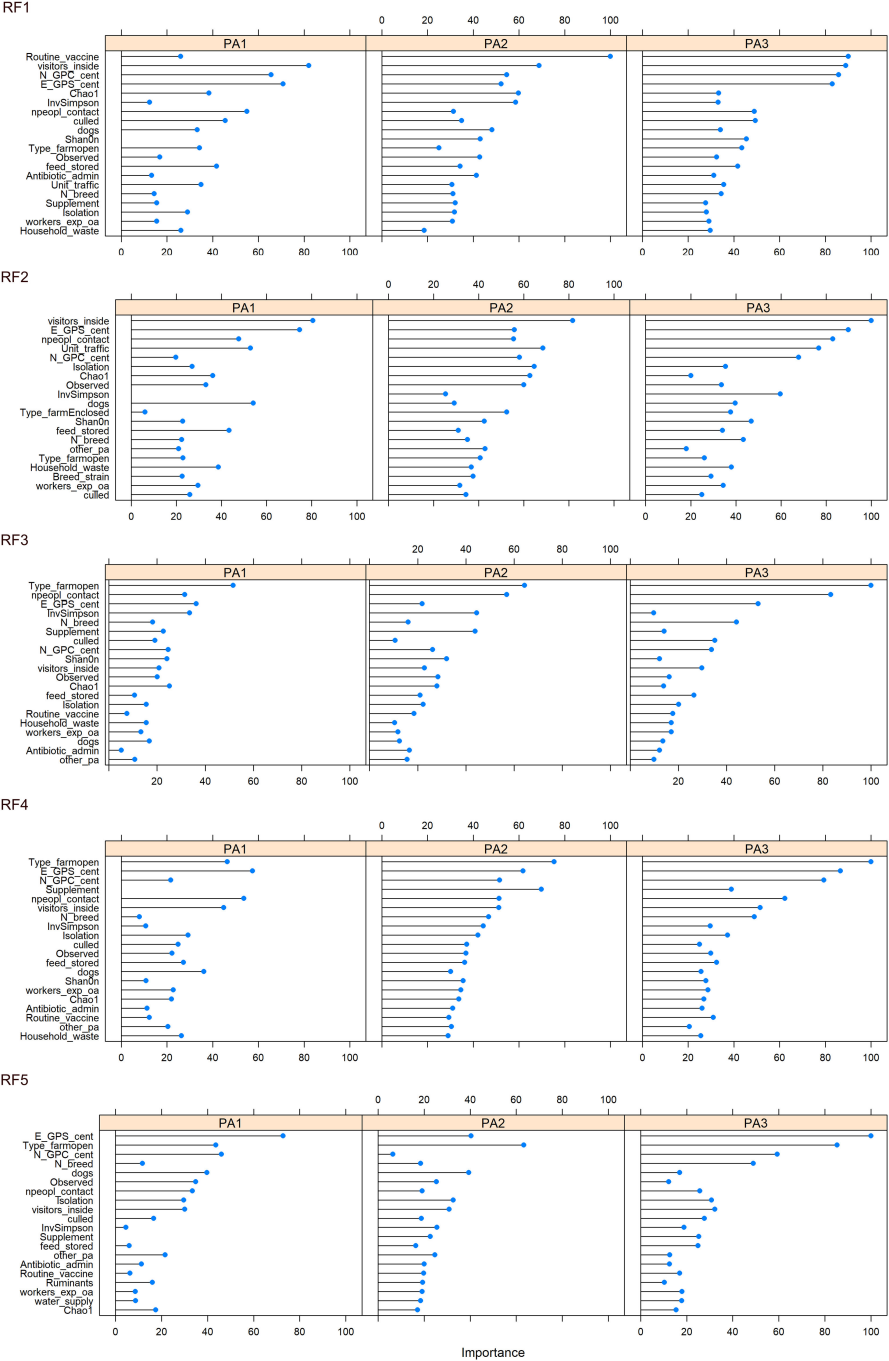
Variable importance for each enterotype, across 5 iterations of the RF model constructed from farming practice, geographical region and alpha diversity measurements. Variable importance indicates which variables contribute the most to the predictive power and accuracy of model.

The top 20 most important variables to classify the data (discriminate between enterotypes) were plotted (**Figure 6**, **Supplementary Data 13**). Across all five RFMs, the location of farms (scaled N and E co-ordinates) appeared to be one of the most important variables (**Supplementary Data 14**). Other variables consistently in the top 10 across all five RFMs were whether visitors were allowed inside and the number of people in contact with the chickens. Four of the RFMs placed importance on the type of chicken lines on farm and three ranked the presence of dogs and whether thinning (i.e. partial flock clearance) took place as important in the top 10. The type of enclosure: open, enclosed or caged was especially important for discriminating PA3, with PA3 almost absent on open farms (Enterotype breakdown for each factor in **Supplementary Data 15**). To discriminate between chickens from the same farm we did not use any data contributing to beta diversity (such as presence or proportion of specific bacteria) as this information is part of what defines the enterotype. However, we did include alpha diversity information, and this consistently came up as an important variable.

The RF models were also run without alpha diversity information, and although this reduced the accuracy and statistical significance, the models still performed better than the NIR, and RF5 was significantly better than the NIR (RF5: p= 0.03683) even without alpha diversity (**Table 2B**).

## 4 Discussion

### 4.1 Microbiomes are complex systems with multiple influences

The chicken caecal microbiome is associated with attributes attractive to poultry producers and consumers, including resistance to pathogen colonisation and disease (McKenna et al., 2020; Patuzzi et al., 2021; Wyszyńska and Godlewska, 2021), efficient food conversion (Huang et al., 2021; Dittoe et al., 2022), high body weight (Xu et al., 2016; Banerjee et al., 2018) and favourable nutrient content (Li et al., 2020). Microbiome composition is shaped by the interaction of multiple influences, from host genetic background to feed, farming characteristics and biosecurity measures (Kers et al., 2018; McKenna et al., 2020). Many studies have investigated the effects of individual factors such as host genetics (breed/line) (Glendinning et al., 2020) and chicken age (Segura-Wang et al., 2021), as well as interventions such as diet (Glendinning et al., 2020), supplements (Li et al., 2020), antibiotics (Costa et al., 2017; Kumar et al., 2018), probiotics (Śliżewska et al., 2020), geography and climate (Pandit et al., 2018; Glendinning et al., 2020; Glendinning et al., 2023), and farming practices (McKenna et al., 2020; Di Marcantonio et al., 2022) on the microbiome (Kers et al., 2018; McKenna et al., 2020; Rashid et al., 2021). However, attempts to investigate these variables individually in experimental comparisons are unlikely to reflect field conditions, as findings based on specific controlled treatments or groupings are difficult to generalise across different systems. Similarly, trying to research the chicken caecal microbiome based on all possible permutations of so many interacting factors is statistically unfeasible.

### 4.2 Enterotypes to simplify and compare microbiome complexity

Our data was complex, coming from 600 chickens belonging to two distinct chicken breeds/lines from 60 farms across a range of locations with different farm management practices. Rather than comparing microbiomes based on variables such as breed/line or diet, we have looked for emergent structure in the microbiomes and defined enterotypes in the data so that the microbiome itself could be our unit of comparison. Enterotypes were first described as “densely populated areas in a multidimensional space of community composition” (Arumugam et al., 2011) and can be viewed as preferred community configurations that tend towards homeostasis, although many variables affect this equilibrium including host health, environment, diet, treatments, and constant exposure to new sources of bacteria (Sommer et al., 2017). Enterotypes are therefore likely to be in constant flux, and microbiomes occur on a spectrum tending towards certain symbiotic optima, rather than as discrete community structures (Jeffery et al., 2012; Costea et al., 2018). Indeed, we found this in our data, which was filtered using several common abundance and prevalence measures to create 18 slight variations of the dataset. By clustering these variations, we identified three consistent clusters (enterotypes) and excluded borderline samples that changed cluster membership between clustering efforts.

Previous methods to detect enterotypes in humans (Arumugam et al., 2011; Mobeen et al., 2018) and chickens (Kaakoush et al., 2014; Yuan et al., 2020; Glendinning et al., 2023) used relative abundance (RA) of count data to cluster samples. We chose to use compositional data (CoDa) approaches to discern enterotypes because it focuses on the ratio between taxa, rather than their relative abundance and is therefore much better at detecting preserved relationships between individual taxa, including low abundance taxa (Gloor et al., 2017). Using PAM clustering of AIT distance, we identified three enterotypes PA1, PA2 and PA3. The identification of three groupings agrees with previous observations in enterotype studies from several other species, including humans(Arumugam et al., 2011; Wu et al., 2011), pigs (Xu et al., 2021a) chimpanzees (Moeller et al., 2012) and chickens (Kaakoush et al., 2014; Yuan et al., 2020; Glendinning et al., 2023). However, the enterotypes identified using a CoDa approach differed from those using traditional approaches such as BC and JSD (**Supplementary Data 8**) that have been used in previous enterotype papers.

A strength of using a compositional data approach and ALDEx2 to detect differential abundance in genera was that this approach was able to highlight several low abundances, rare taxa that might play a significant role in determining the microbial community enterotype. The PA3 enterotype exhibited the least overlap with PA1 and PA2 (PCA biplot and ALDEx2 results). Consideration of the differentially abundant taxa in the PA3 enterotype revealed most belonged to rare taxa. The PA3 enterotype also had the strongest bacterial networks, with strong evidence of symbiotic reliance, and yet the genera making up these network clusters were exceedingly rare, and very little functional information is known about them.

Although our measures of internal cluster validity were low, (below the minimum 0.2 SI value recommended to suggest structure and lying just above the limit of detection from random noise), it was comparable to previous studies on gut enterotypes that had similarly low cluster support with silhouette values less than 0.2 (Moeller et al., 2012; Koren et al., 2013). This is not unexpected in a complex phenotype that lies on a continuous scale in multidimensional space.

### 4.3 Enterotypes can be further simplified into interacting guilds

Bacteria that are highly connected with positive associations in co-occurrence networks are thought to represent ecological guilds because they thrive or decline together depending on resource abundance (Wu et al., 2021; Wu et al., 2022). Our network analysis revealed structure to bacterial co-occurrence relationships that was evident across all three enterotypes. In all cases, networks could be divided into guilds, where bacteria within each guild were highly connected, with a negative correlation between guilds. One of these guilds consisted mainly of Firmicutes, and was inversely correlated to the second guild, consisting of Bacteroides, Proteobacteria and others.

The growing awareness of microbial guilds in gut microbiomes has led to the suggestion of replacing the concept of enterotypes with enterosignatures. Each guild represents an enterosignature and the microbiome can be described in terms of the proportion of enterosignatures/guilds (Frioux et al., 2023). Interestingly, enterotypes and enterosignatures were found to correlate strongly, with the enterotype generally being classified as the most dominant enterosignature or guild. This agrees with our observation that our enterotypes were either Firmicutes-dominant (PA1) or Bacteroides-dominant (PA3). A “seesaw” network between Firmicutes-dominant and Bacteroides-dominant guilds has previously been explicitly described in human gut microbiomes (Wu et al., 2022).

### 4.4 Characteristics of the three caecal enterotypes

#### 4.4.1 Enterotypes and *Campylobacter* abundance

A major application of studying enterotypes is to apply insights on differential community compositions to stage positive public health interventions. The most significant finding of this study was therefore the significant differential abundance of *Campylobacter* in the different enterotypes. The abundance of both *Campylobacter* and *E.coli* was significantly increased in PA2 compared to other enterotypes (**Figure 3**) and *E.coli* and *Campylobacter* were found to be positively correlated in a Network analysis (**Figure 5**). Although *Campylobacter* was also abundant in PA3 based purely on comparisons of RA (**Figure 3E**), ALDEx2 and LEfSe did not identify this increase as significant. This may be because the differences in *Campylobacter* abundance between PA1 and PA3 are still dwarfed compared in comparison to the differences in abundance of other genera between PA1 and PA3. However, the risk to public health from *Campylobacter* in PA3 may be similar between PA1 and PA3 due to its abundance irrespective of the different community dynamics that drive the abundance.

*Campylobacter* has been associated with increases in Proteobacteria and Bacteroidota in several studies (Dicksved et al., 2014; Pang et al., 2023). In a study of faecal microbiomes of poultry abattoir workers, *Campylobacter* culture-positive individuals had significantly higher Bacteroides and *Escherichia* (Proteobacteria) species than those who remained culture negative. This would agree with our observations of a direct positive correlation with *E.coli* (a Proteobacteria) in PA2 and with the high abundance of *Campylobacter* in PA3- the enterotype with the lowest F:B ratio and dominated by a Bacteroidota and Proteobacterial guild in network analysis. Furthermore, several studies have shown significant positive associations between *Campylobacter* and *Phascolarctobacterium* (Dicksved et al., 2014; Pang et al., 2023). Indeed, we noted the same relationship between *Campylobacter* and *Phascolartobacterium* in our PA1 network analysis, however in this case, *Phascolartobacterium* had significantly lower abundance and was negatively correlated with the highly abundant Firmicutes-dominant guild. Although we did not detect a direct correlation between *Phascolartobacterium* and *Campylobacter* in our PA2 network analysis, *Phascolatrtobacterium* was both the most abundant and the most differentially abundant genus in PA2.

#### 4.4.2 Differences in alpha diversity

PA1 and PA2 were more alike than either was to PA3, however they had a significant difference in their alpha diversity (**Figures 2D–G**). This is interesting because there is a relationship between high species diversity and ecosystem stability known as the insurance hypothesis (Sommer et al., 2017). This hypothesis suggests that microbiome resilience occurs in highly diverse communities because several species compete for the limited resources, thus limiting the influx or overgrowth of other species (Sommer et al., 2017). High alpha diversity microbiomes are therefore more stable, whereas a community with low alpha diversity is less resilient and more likely to tip into dysbiosis in response to perturbation. The propensity for low alpha diversity to reflect a vulnerability towards dysbiosis is suggested by its association with several human diseases, including chronic autoimmune conditions of the gut (Ott et al., 2004; Manichanh et al., 2006; Willing et al., 2010), recurrent *Clostridium difficile* infection (Chang et al., 2008) and even increased frailty in old age (Jackson et al., 2016).

Because PA1 and PA2 were so similar, it is tempting to speculate that PA2 may represent a subset of enterotype PA1 that is tending to dysbiosis. It is difficult to know whether the low alpha diversity enables the proliferation of *Campylobacter*, or if alpha diversity drops in response to *Campylobacter* proliferation. However, a relationship between low caecal alpha diversity, high *Campylobacter* abundance and low welfare poultry farms has been observed (Di Marcantonio et al., 2022), suggesting that low alpha diversity and high *Campylobacter* may represent a stressed state. Furthermore, because all birds in this study were apparently healthy at sampling, any changes towards dysbiosis are expected to be minor. Further investigations to compare the microbiomes of healthy and truly dysbiotic birds will help to clarify the significance of alpha diversity and caecal gut health.

#### 4.4.3 F:B ratio differences across enterotypes

The major gradient in the gut microbiome of many species is the ratio of Firmicutes to Bacteroides (F:B) and we noted a difference in this ratio across PA1, PA2 and PA3, with PA1 having the highest F:B ratio and PA3 having the lowest (**Figure 2A**). Diet may play a role in determining F:B ratio, as members of these phyla have complementary but distinct metabolic capacities (Flint et al., 2012; O. Sheridan et al., 2016; Ali et al., 2022; Frioux et al., 2023). Bacteroidota have a high number of CAZymes against animal and plant cell wall carbohydrates but are poor metabolizers of alpha-glucans (Frioux et al., 2023). Alpha-glucans, present in starch, represent a high proportion of the energy composition in poultry feed (Svihus 2014). Many Firmicutes can metabolize alpha-glucans and this may explain their abundance in commercial farm broilers. A high F:B ratio has previously been associated with improved food conversion efficiency and higher body weight (Singh et al., 2013; Xu et al., 2016; Banerjee et al., 2018). For example, the use of antibiotic growth promoters results in an increase in F:B ratio and improved productivity (Banerjee et al., 2018). The effect of F:B ratio on *Campylobacter* abundance is mixed. A small study (n=2) identified a decreased F:B ratio in a chicken caecum after *Campylobacter* challenge (Qu et al., 2008), while a study of 100 chickens from 4 farms found no association with F:B ratio (Sakaridis et al., 2018) and study of caeca from 10 chickens showed a non-significant trend between high F:B ratio and low *Campylobacte*r abundance (Sofka et al., 2015). In this study, a high F:B ratio (PA1) was correlated with low *Campylobacter* abundance.

PA1 had a higher proportion of butyrate producers (Ruminococcacea), and several genera, such as *Faecalibacterium* that are associated with good gut health (Hornef and Pabst, 2016; Lopez-Siles et al., 2017). Butyrate producers like *Faecalibacterium* are associated with low inflammation for two reasons. Firstly, their presence provides health benefits: when butyric acid is sensed by the host, the host responds by strengthening the epithelial barrier, reducing inflammation, and increasing the production of mucins and antimicrobial peptide (Onrust et al., 2015; Hornef and Pabst, 2016). Their reduction in the gut may signal the presence of inflammation, as the vegetative cells of buyrate-producing Ruminococcacea and Lachnospiracea are extremely sensitive to the presence of oxygen, and so their absence often indicates the presence of reactive oxygen species (ROS) from macrophages and granulocytes (Rychlik, 2020). In chickens, *Faecalibacterium* is significantly less abundant in chickens infected with *Eimeria tenella*, than in controls(Yu et al., 2023). In fact, there is significantly lower *Faecalibacterium* abundance in frail patients (Jackson et al., 2016) and patients with inflammatory bowel disease (Sokol et al., 2009). Secondly, *Faecalibacterium* may play a role in promoting temporal stability, or resilience of the gut (Olsson et al., 2022).

PA3 had relatively high *Campylobacter* abundance but was particularly interesting as it had a high abundance of several genera that were absent or low abundance in other enterotypes. For example, PA3 had the highest abundance of *Mucispirillum schaedleri*. In mice, high abundance of *M. schaedleri*, a member of the phylum Deferribacteres, associated with significantly reduced gut inflammation compared with control animals following infections with *Salmonella* Typhimurium, even when pathogen loads in the gut were unchanged (Du Toit, 2019).

### 4.5 Farm factors affecting enterotype

An understanding of enterotypes is only truly useful when we can harness our understanding to manipulate enterotypes to stage public health interventions. To do this, we need to understand the factors that determine enterotype composition. As previously mentioned, the microbiome is influenced by multiple interacting factors. Inter-farm variation is often higher than intra-farm variation for several obvious reasons. For example, chickens on the same farm are often genetically related, or of the same breed/line, share similar geography and farming practices. The proximity of chickens to each other within a farm increases the likelihood of inoculation and sharing of bacterial species between members of the same flock (Sakaridis et al., 2018). Thus, we found that chickens from the same farm were more likely to share an enterotype than chickens from different farms, even when both breeds/lines were represented.

Random Forest Models (RFMs) have previously been developed to predict the prevalence of pathogens such as *Listeria* spp (Golden et al., 2019) and *Campylobacter* (Xu et al., 2021b) from farm practice data. An RFM was not able to predict *Campylobacter* prevalence from this data, because prevalence across all samples was too high. In this study, 100% of farms had at least one chicken with *Campylobacter*, and the total number of birds with *Campylobacter* detected in their caecal microbiome was 455/600. However, it was possible to use a RFM model to detect enterotype from farm practice metadata because there were only three enterotypes and multiple permutations of variables.

Geography (scaled N and E co-ordinates) appeared as one of the most important variables to discriminate between enterotypes (**Supplementary Data 15** shows the distribution of enterotypes across farms). A pilot study on these chicken lines previously highlighted a strong effect of geography on the chicken microbiome (Pandit et al., 2018) and a recent study of scavenging indigenous chickens in Ethiopia also clustered the caecal microbiome into three enterotypes- which were found to largely be influenced by geographic factors like altitude, climate, and topsoil (Glendinning et al., 2023).

However, apart from carefully choosing the location of your farm, major interventions to prevent pathogen colonisation in poultry can be made via farming management practices (Sibanda et al., 2018; Soro et al., 2020). Stringent biosecurity measures are required to prevent the introduction of *Campylobacter*, since the infection of a single chicken can rapidly result in the colonisation of an entire flock within a week (Evans and Sayers, 2000). Nevertheless, farms with high biosecurity measures still routinely have flock colonisation (Sibanda et al., 2018). The most useful distinction may be to understand the factors that result in enterotype PA1, which had a significantly lower campylobacter abundance than PA2 or PA3.

Because PA2 was associated with high *Campylobacter* abundance and PA1 was associated with low *Campylobacte*r abundance, it may be possible to identify the environmental factors and farming practices that contribute to that enterotype. Modifiable factors that influence enterotype identity could be used to reduce farm-level abundance of zoonotic pathogens such as *Campylobacter*, decreasing the risk of subsequent human exposure and disease. The main factors that differed between enterotype PA1 and PA2 were the number of people in contact, the presence of dogs, whether visitors were allowed inside and routine vaccination (**Figure 6**).

### 4.6 Future work

Finally, there were limitations in this study. First, although 60 farms and 600 chickens represent a decent sized dataset, we would have benefited from a greater range of samples from more farms. Additional limitations related to identifying enterotypes include the taxonomic resolution of the study, the number of species representatives in current databases, sequencing depth, and filtering thresholds. ASVs were agglomerated at genus level, and occasionally at even higher levels where sequences could only be assigned to higher ranks such as family, and even order. The number of genera of interest in this study that either have no cultured relatives or could not be classified to genus level highlights the vast gaps in knowledge about bacteria inhabiting the chicken digestive tract. Some Lachnospirae, Ruminococcaceae, Oscillospiraceae and Gastranaerophilaceae could only be identified at family level, and Christensenellales at order level. This undoubtably affected detection of significant functional differences between genera from these groups in the different enterotypes. The rate at which this limitation is being addressed is heartening, with three excellent metagenomics studies of the chicken caecum recently adding thousands of novel genomes to the GTDB database (Glendinning et al., 2020; Gilroy et al., 2021; Segura-Wang et al., 2021). However, none of these recent metagenomic surveys included chickens from India, or these specific breeds/lines. While bacteria in these chickens are likely to be similar, there may be differences linked to factors such as the genetics of the indigenous Kadaknath chickens as well as local farming practices including the use, dosages and classes of antibiotics administered. A truly representative dataset of chicken microbial genomes for taxonomic classification should include sequences of caecal samples from multiple genetic background derived from global locations, raised under a variety of conditions (Glendinning et al., 2020).

## 5 Conclusions

Campylobacteriosis is a serious cause of human gastrointestinal disease, and poultry is a major source. *Campylobacter* is highly prevalent in most chickens, but its abundance is affected by farming practices, biosecurity measures and microbiome composition. We found that the caecal microbiome of chickens can be split into three enterotypes that occur on a gradient and differ in their proportion of Firmicutes and Bacteroides. PA1 appears to represent a resilient phenotype, with high F:B ratio, a low abundance of *Campylobacter* and a high abundance of *Faecalibacterium*. Enterotype PA2 had the lowest F:B ratio, lower alpha diversity, and the highest abundance of *Campylobacter.* We speculate that PA2 represents a version of PA1 that is tending towards dysbiosis. PA3 was significantly different from the other two enterotypes and had both high alpha diversity, high *Campylobacter* load and several genera that were absent in the other enterotypes. Random Forest Models were able to predict the enterotype of individual birds based on farming characteristics and alpha diversity. Together, this suggests that we may be able to identify farming practices that affect enterotype and the microbial community interactions within enterotypes that explain the differences in *Campylobacter* abundance and mechanisms behind resilient and susceptible enterotypes.

## Data availability statement

The 16S rRNA gene sequence data has been uploaded on EBI-ENA under Project ID PRJEB15343, SRA ID ERP017060. In addition, all scripts used to analyze the data and generate the figures are available at https://github.com/MelanieCHay/C-enterotypes.

## Ethics statement

The animal studies were approved by Ethical Review Panel of Anand Agricultural University (AAU) and the Clinical Research Ethical Review Board (CRERB) of the Royal Veterinary College under the reference URN 2014 1280. The studies were conducted in accordance with the local legislation and institutional requirements. Written informed consent was obtained from the owners for the participation of their animals in this study.

## Author contributions

MH: conceptualization, formal analysis, investigation, methodology, visualization, writing – original draft, data curation. AH: data curation, formal analysis, investigation, methodology, writing – review & editing. RP: investigation, writing – review & editing, data curation, methodology, validation. PK: investigation, methodology, writing – review & editing, conceptualization, funding acquisition, project administration, resources, supervision. P-YL: conceptualization, writing – review & editing. MP: data curation, investigation, writing – review & editing. SJ: data curation, investigation, methodology, writing – review & editing, validation. XD: conceptualization, writing – review & editing. MC: data curation, investigation, writing – review & editing. BF: investigation, methodology, writing – review & editing, data curation. GL: data curation, writing – review & editing, investigation. JG: data curation, investigation, writing – review & editing. DX: conceptualization, writing – review & editing. FT: conceptualization, funding acquisition, investigation, project administration, resources, writing – review & editing. CJ: conceptualization, funding acquisition, investigation, project administration, resources, writing – review & editing. AP: conceptualization, funding acquisition, investigation, project administration, resources, writing – review & editing. DB: conceptualization, formal analysis, funding acquisition, investigation, methodology, project administration, resources, supervision, writing – original draft, writing – review & editing.

## Supplementary material

Supplementary Data Sheet 1 Table of geographical location and farming practices of the 60 farms.

Supplementary Data Sheet 2 Datasets filtered by prevalence and abundance and variation explained by each dataset.

Supplementary Data Sheet 3 Comparison of clusters using different distance and clustering methods.

Supplementary Data Sheet 4 Random Forest: table of variables tested, and levels per variable.

Supplementary Data Sheet 5 Boxplots of summary statistics to remove low diversity outlier samples.

Supplementary Data Sheet 6 Detecting optimal number of clusters for PAM clustering of Aitchinson distance across 18 datasets.

Supplementary Data Sheet 7 Cluster memberships of samples by PAM-clustered Aitchison distance across the 18 datasets + cluster intersects.

Supplementary Data Sheet 8 PHATE comparison of count data and clr data for AIT, BC and JSD.

Supplementary Data Sheet 9 Tables of mean relative abundance of Phyla and Genera for each of the three enterotypes.

Supplementary Data Sheet 10 Excel with ALDEx2 results for PA1 vs PA2, PA1 vs PA3, PA2 vs PA3 and Full ALDEx2 plots.

Supplementary Data Sheet 11 Network comparisons for PA1 vs PA2, PA1 vs PA3 and PA2 vs PA3. Network only includes genera that are present in both enterotypes being compared.

Supplementary Data Sheet 12 Farms belonging to different data partitions for each of the 5 Random Forest Models.

Supplementary Data Sheet 13 Important variables for each enterotype over the five Random Forest iterations.

Supplementary Data Sheet 14 Map showing distribution of enterotypes across farms in Gujarat.

Supplementary Data Sheet 15 Proportion of enterotypes of each of the farm characteristics.
