# Supplementary material for: Chicken caecal enterotypes in indigenous Kadaknath and commercial Cobb chicken lines are associated with Campylobacter abundance and influenced by farming practices
Source: Front Microbiomes. 2023 Dec 4;2:1301609. doi: 10.3389/frmbi.2023.1301609 (PMC12993513; doi:10.3389/frmbi.2023.1301609)
Supplement: Supplementary Data Sheet 1 — Table of geographical location and farming practices of the 60 farms. [file DataSheet_1.zip › Supplementary Data 2.docx]

**Table 1. Table showing the effect of prevalence and abundance filtering criteria on the number of taxa (genera), library size and ability to explain variation (PCA components).**

|  |  |  |  |  |  |  | **PCA** | | | | | | **prcomp** | | | | | | **PCA** | | | | | |
| --- | --- | --- | --- | --- | --- | --- | --- | --- | --- | --- | --- | --- | --- | --- | --- | --- | --- | --- | --- | --- | --- | --- | --- | --- |
| **Prevalence** | | **Abundance** | |  |  |  | **Abundance ranks PCA** | | | | |  | **Compositional PCA** | | | | | | **Compositional PCA** | | | | | |
| **no samples** | **%** | **RA level** | **Filt (%)** | **N**  **taxa** | **% reads** | **N reads** | **PC1** | **PC2** | **PC3** | **PC4** | **PC5** | **Total** | **PC1** | **PC2** | **PC3** | **PC4** | **PC5** | **Total** | **PC1** | **PC2** | **PC3** | **PC4** | **PC5** | **Total** |
| 0 | 0 | 0 | 0 | 625 | 100% | 46,204,478 |  |  |  |  |  |  |  |  |  |  |  |  |  |  |  |  |  |  |
| 6 | 1 | 0.001 | 0.1 | 232/ | 99.66 | 46,049,079 | 12.53 | 6.25 | 4.09 | 3.74 | 3.45 | 30.06 | 12.2 | 6.3 | 4.5 | 3.6 | 3.3 | 29.9 | 12.4 | 6.6 | 4.3 | 3.8 | 3.4 | 30.5 |
| 6 | 1 | 0.0001 | 0.01 | 329 | 99.96 | 46,187,839 | 13.02 | 7.58 | 4.48 | 3.06 | 2.52 | 30.66 | 10.6 | 6.9 | 4.7 | 3.6 | 3.2 | 29 | 11.3 | 7.3 | 4.8 | 3.1 | 2.9 | 29.4 |
| 6 | 1 | 0.00001 | 0.001 | 381 | 99.98 | 46,193,646 | 16.47 | 6.98 | 4.19 | 2.72 | 2.16 | 32.52 | 10.4 | 8 | 4.6 | 3.6 | 3.2 | 29.8 | 13.2 | 7.1 | 4.5 | 2.9 | 2.5 | 30.2 |
| 12 | 2 | 0.001 | 0.1 | 209 | 99.45 | 45,949,179 | 12.33 | 5.92 | 4.34 | 3.69 | 3.58 | 29.86 | 12.4 | 6.1 | 4.7 | 3.8 | 3.1 | 30.1 | 12.1 | 6.3 | 4.7 | 3.8 | 3.5 | 30.4 |
| 12 | 2 | 0.0001 | 0.01 | 302 | 99.93 | 46,171,076 | 11.9 | 7.3 | 4.6 | 3.3 | 2.7 | 29.8 | 10.9 | 6.3 | 4.7 | 3.5 | 3.3 | 28.7 | 11 | 6.8 | 4.8 | 3.3 | 3 | 28.9 |
| 12 | 2 | 0.00001 | 0.001 | 345 | 99.96 | 46,184,973 | 13.9 | 7.5 | 4.4 | 2.9 | 2.4 | 31.1 | 10.5 | 7.2 | 4.7 | 3.6 | 3.2 | 29.2 | 11.3 | 7.3 | 4.8 | 3.1 | 2.7 | 29.2 |
| 24 | 4 | 0.001 | 0.1 | 191 | 99.01 | 45,745,752 | 12.5 | 5.9 | 4.6 | 3.7 | 3.5 | 30.2 | 12.6 | 6 | 4.9 | 3.9 | 3 | 30.4 | 12.3 | 6.3 | 4.9 | 3.9 | 3.5 | 30.9 |
| 24 | 4 | 0.0001 | 0.01 | 265 | 99.79 | 46,106,260 | 11.4 | 6.5 | 4.7 | 3.5 | 3 | 29.1 | 11.3 | 5.9 | 4.7 | 3.4 | 3.2 | 28.5 | 11 | 6.1 | 4.8 | 3.4 | 3.1 | 28.4 |
| 24 | 4 | 0.00001 | 0.001 | 297 | 99.89 | 46,152,021 | 11.5 | 7.5 | 4.6 | 3.3 | 2.8 | 29.7 | 10.8 | 6.2 | 4.7 | 3.4 | 3.3 | 28.4 | 10.5 | 6.6 | 4.9 | 3.2 | 3.1 | 28.3 |
| 30 | 5 | 0.001 | 0.1 | 174 | 98.19 | 45,366,415 | 12.6 | 6.1 | 4.9 | 3.9 | 3.3 | 30.8 | 12.9 | 6.1 | 5 | 4.1 | 2.9 | 31 | 12.4 | 6.5 | 5.1 | 4 | 3.4 | 31.4 |
| 30 | 5 | 0.0001 | 0.01 | 250 | 99.62 | 46,028,259 | 11.5 | 6.1 | 4.6 | 3.6 | 3.2 | 29 | 11.5 | 5.9 | 4.6 | 3.5 | 3 | 28.5 | 11.1 | 6 | 4.7 | 3.5 | 3.1 | 28.4 |
| 30 | 5 | 0.00001 | 0.001 | 283 | 99.81 | 46,115,443 | 11.2 | 7.2 | 4.7 | 3.4 | 2.9 | 29.4 | 10.9 | 6 | 4.7 | 3.4 | 3.3 | 28.3 | 10.5 | 6.3 | 4.9 | 3.3 | 3.1 | 28.1 |
| 60 | 10 | 0.001 | 0.1 | 139 | 96.32 | 44,502,734 | 13.1 | 6.8 | 5.4 | 4.2 | 3.1 | 32.6 | 13.9 | 6.6 | 5.5 | 4.3 | 3.1 | 33.4 | 13.2 | 7 | 5.3 | 4.2 | 3.5 | 33.2 |
| 60 | 10 | 0.0001 | 0.01 | 221 | 98.96 | 45,725,151 | 11.2 | 6 | 4.2 | 3.6 | 3.5 | 28.5 | 11.7 | 5.8 | 4.6 | 3.7 | 2.6 | 28.4 | 10.7 | 5.9 | 4.7 | 3.6 | 3.3 | 28.2 |
| 60 | 10 | 0.00001 | 0.001 | 242 | 99.40 | 45,929,359 | 10.8 | 6.2 | 4.4 | 3.8 | 3.2 | 28.4 | 11.2 | 5.7 | 4.4 | 3.6 | 2.7 | 27.6 | 10.4 | 5.8 | 4.5 | 3.5 | 3.1 | 27.3 |
| 90 | 15 | 0.001 | 0.1 | 124 | 94.39 | 43,613,812 | 12.3 | 7.1 | 5.3 | 4.6 | 3.4 | 32.7 | 13.6 | 7 | 5.5 | 4.8 | 3.3 | 34.2 | 12.9 | 7.4 | 5.2 | 4.6 | 3.8 | 33.9 |
| 90 | 15 | 0.0001 | 0.01 | 199 | 98.50 | 45,510,924 | 11.2 | 5.8 | 4.4 | 3.7 | 3.6 | 28.7 | 11.9 | 5.6 | 4.7 | 3.9 | 2.7 | 28.8 | 10.7 | 5.8 | 4.9 | 3.7 | 3.4 | 28.5 |
| 90 | 15 | 0.00001 | 0.001 | 223 | 98.75 | 45,628,157 | 10.5 | 6.1 | 4.4 | 4 | 3.4 | 28.4 | 11.2 | 5.6 | 4.5 | 3.6 | 2.6 | 27.5 | 10.1 | 5.7 | 4.6 | 3.5 | 3.2 | 27.1 |
