# Supplementary material for: Chicken caecal enterotypes in indigenous Kadaknath and commercial Cobb chicken lines are associated with Campylobacter abundance and influenced by farming practices
Source: Front Microbiomes. 2023 Dec 4;2:1301609. doi: 10.3389/frmbi.2023.1301609 (PMC12993513; doi:10.3389/frmbi.2023.1301609)
Supplement: Supplementary Data Sheet 1 — Table of geographical location and farming practices of the 60 farms. [file DataSheet_1.zip › Supplementary Data 5.pdf]

Determining outliers.

The data was explored first to detect outlier samples. Alpha diversity was used to flag samples that were extreme or aberrant compared to the majority. Descriptive statistics on measures of alpha diversity (Observed ASVs, Shannnon Diversity, InvSimspon and Chao1) on the full dataset before and after rarefying were compared. After noting a bimodal distribution in alpha diversity measures, the dataset was split into Kadaknath and Commercial broiler prior to descriptive statistics.

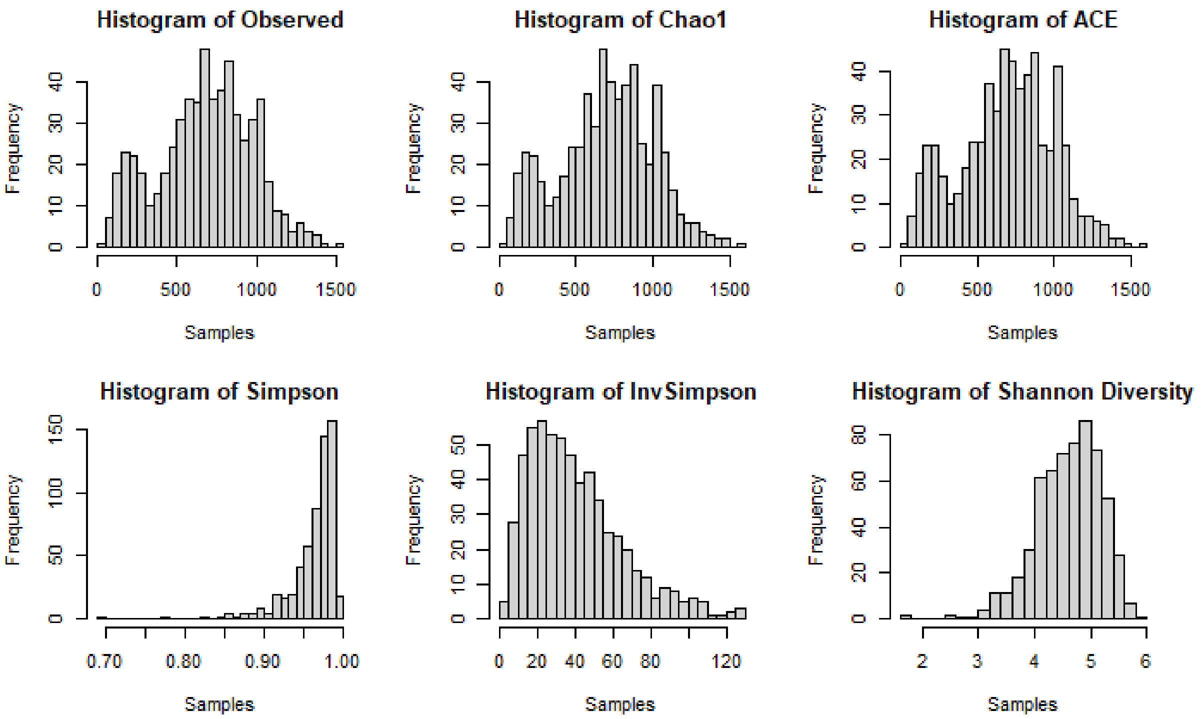

Figure 1Histogram of alpha diversity measures on unrarefied dataset, with both breeds combined shows that there is a bimodal distribution.

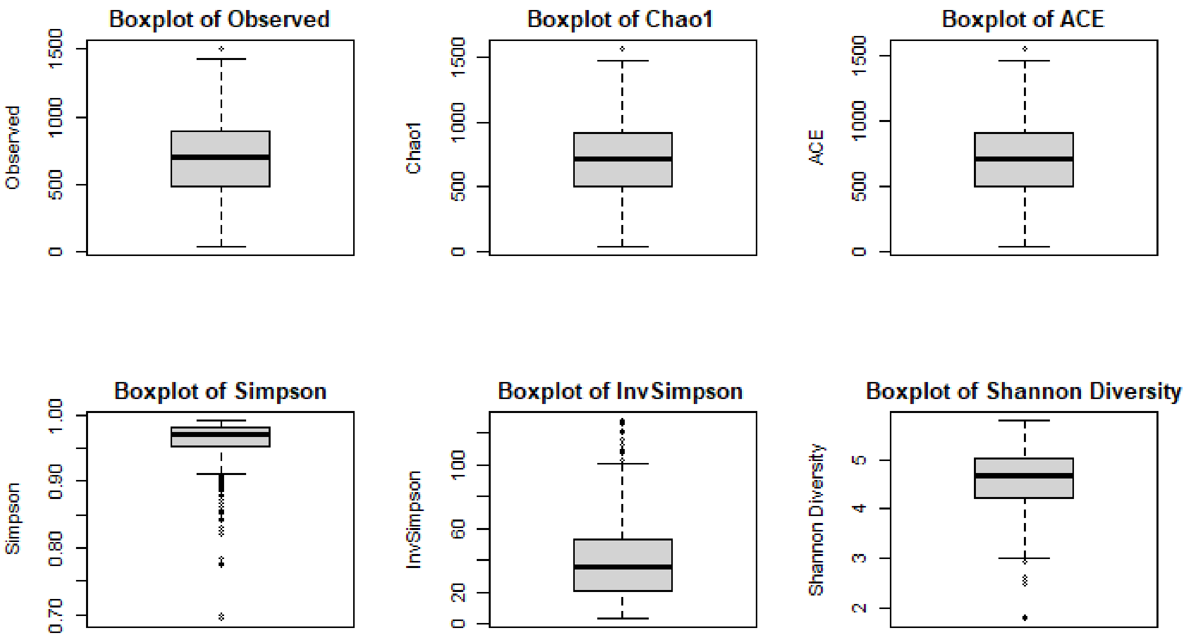

Figure 2 Boxplot of alpha diversity on unrarefied dataset, with breeds combined.

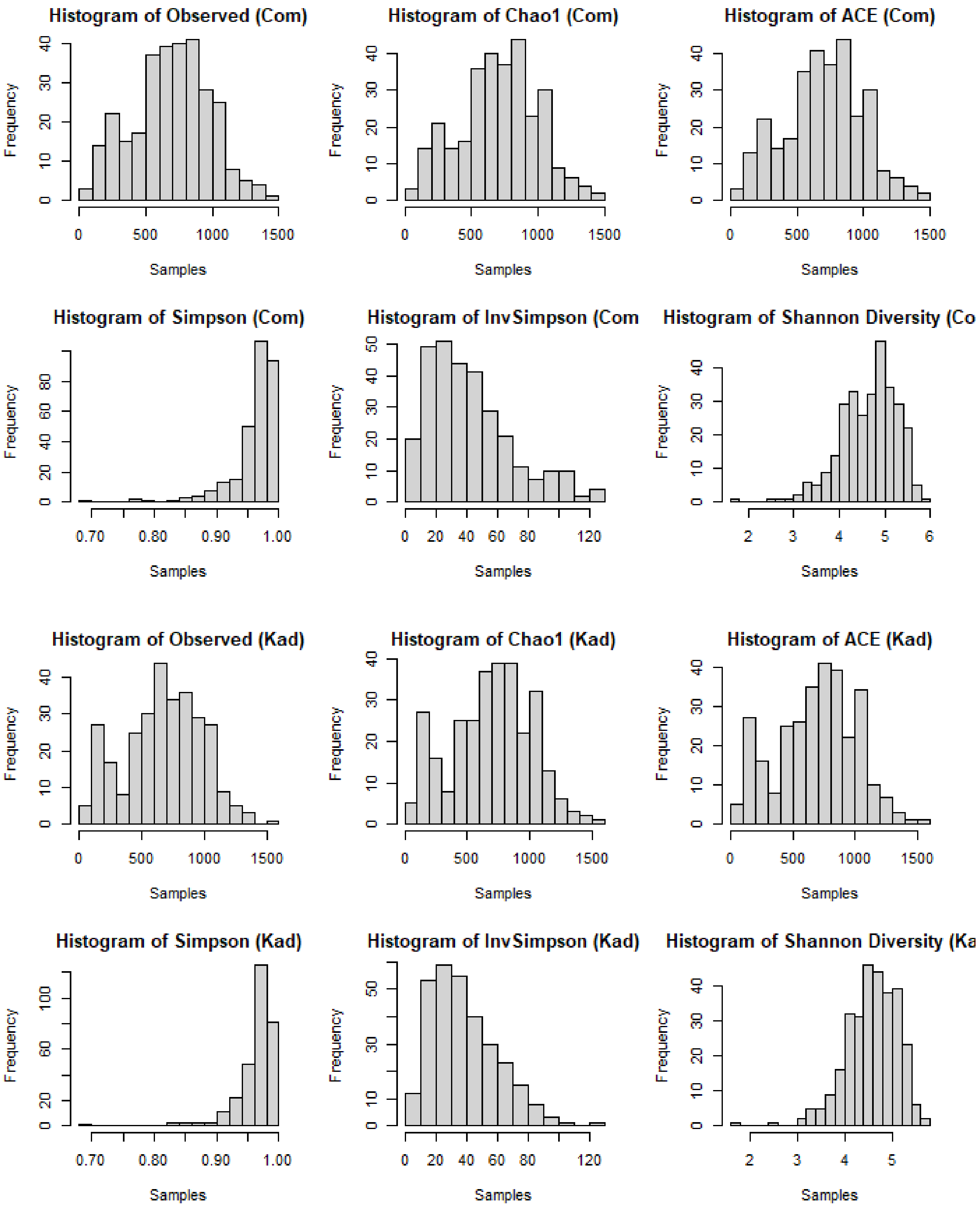

Histogram of Observed (Com)
Histogram of Chao1 (Com)
Histogram of ACE (Com)
Histogram of Simpson (Com)
Histogram of InvSimpson (Com
Histogram of Shannon Diversity (Co
Histogram of Observed (Kad)
Histogram of Chao1 (Kad)
Histogram of ACE (Kad)
Histogram of Simpson (Kad)
Histogram of InvSimpson (Kad)
Histogram of Shannon Diversity (Ka
Frequency
Samples

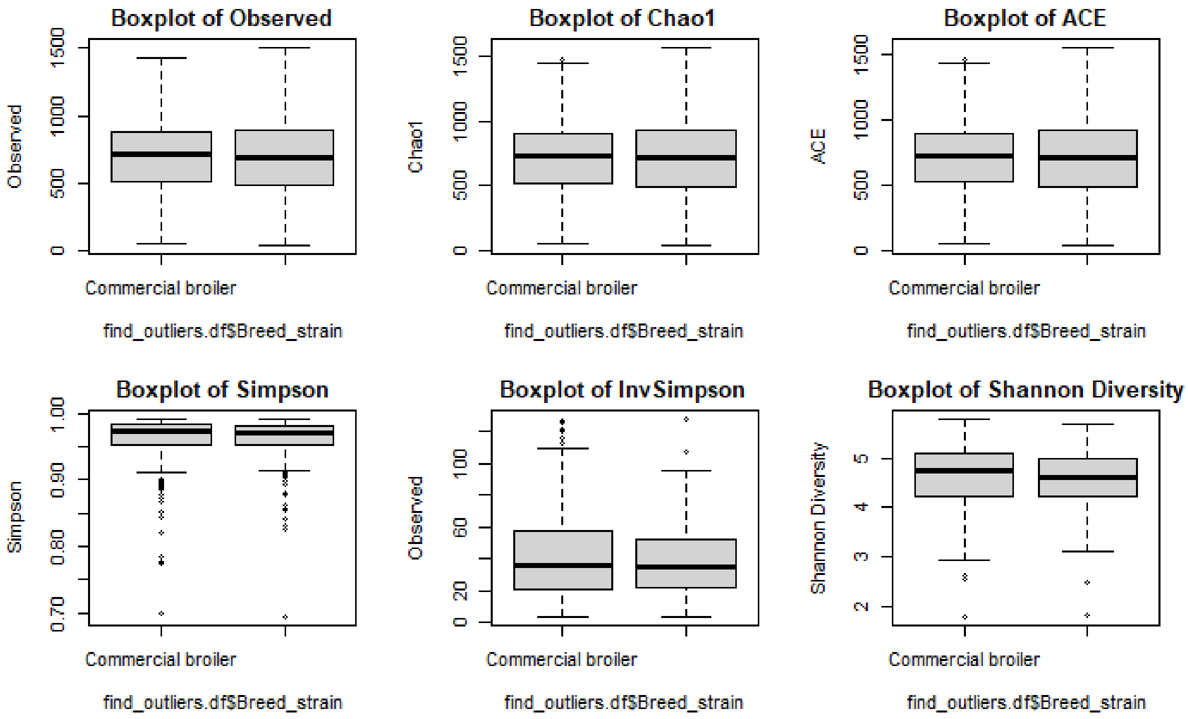

Figure 3 Boxplot of alpha diversity measures on unrarefied datatset, with samples split by breed.

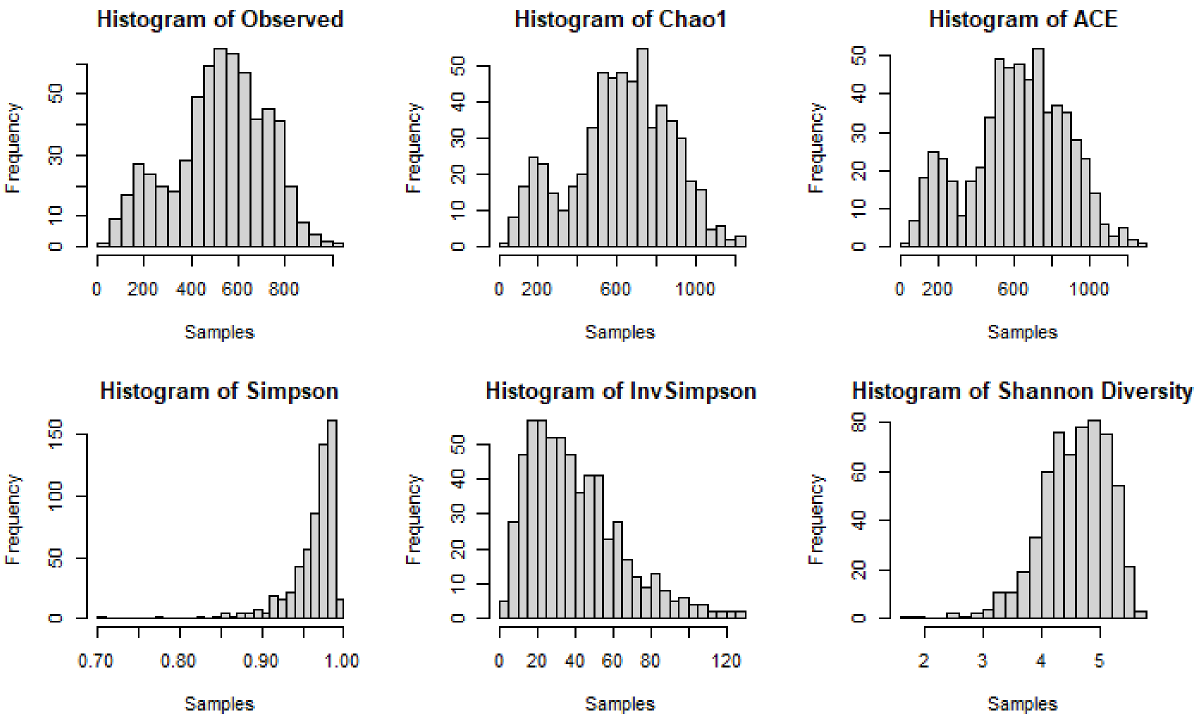

Figure 4 Histogram of alpha diversity measures on rarefied, combined datatset.

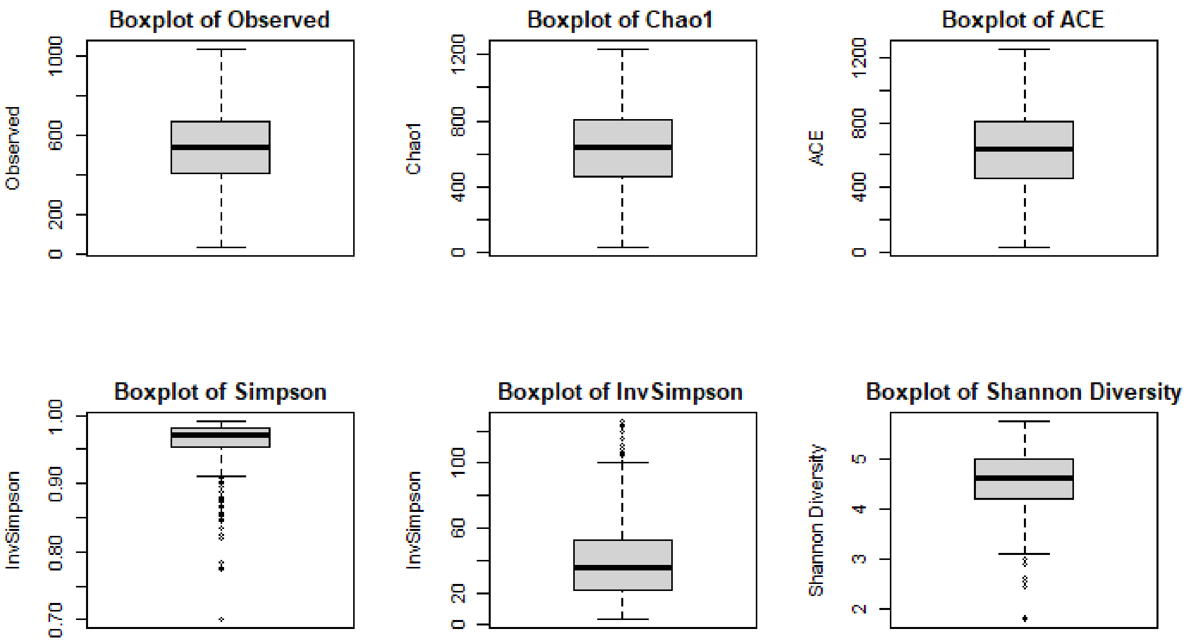

Figure 5 Boxplot of alpha diversity measures on rarefied, combined dataset.
