# Supplementary material for: Chicken caecal enterotypes in indigenous Kadaknath and commercial Cobb chicken lines are associated with Campylobacter abundance and influenced by farming practices
Source: Front Microbiomes. 2023 Dec 4;2:1301609. doi: 10.3389/frmbi.2023.1301609 (PMC12993513; doi:10.3389/frmbi.2023.1301609)
Supplement: Supplementary Data Sheet 1 — Table of geographical location and farming practices of the 60 farms. [file DataSheet_1.zip › Supplementary Data 8.pdf]

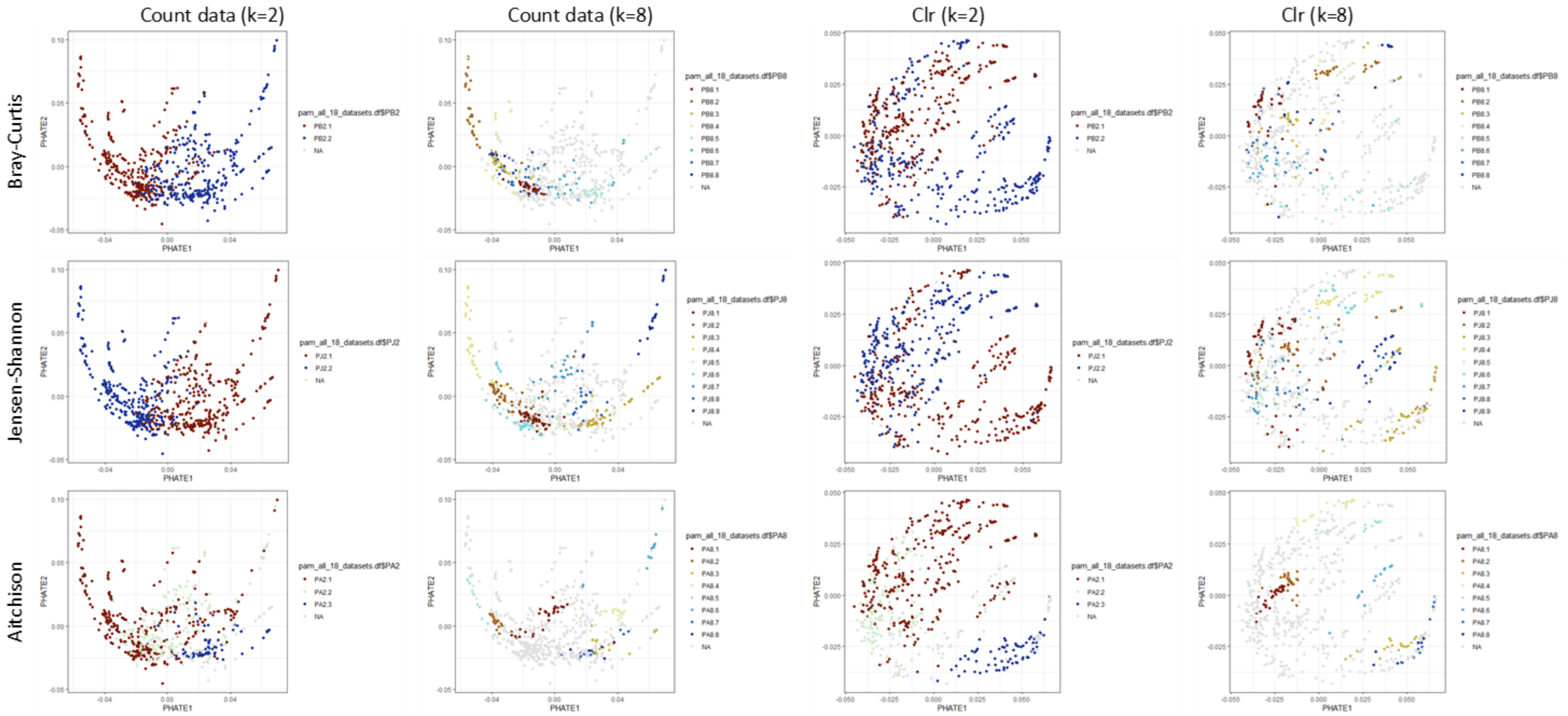

Supplementary Figure 8. Visualisation of cluster assignment use PhateR: Figure shows spread of count data and clr data for the dataset with the lowest filtering thresholds (prevalence = 1%, RA = 0.001%, genera = 381), with points coloured by cluster assignment. Clusters were created by PAM clustering based on Bray-Curtis, Jensen Shannon Divergence and Aitchison distance using k=2 and k=8 clusters (determined by silhouette method to be the optimal number of clusters). Clusters are the consensus results from the intersection of 18 different filtered datasets (table xx). NA (in grey) reflects samples that were assigned to conflicting clusters in the different filtered datasets.
