# Supplementary figures and images for: Chicken caecal enterotypes in indigenous Kadaknath and commercial Cobb chicken lines are associated with Campylobacter abundance and influenced by farming practices
Source: Front Microbiomes. 2023 Dec 4;2:1301609. doi: 10.3389/frmbi.2023.1301609 (PMC12993513; doi:10.3389/frmbi.2023.1301609)

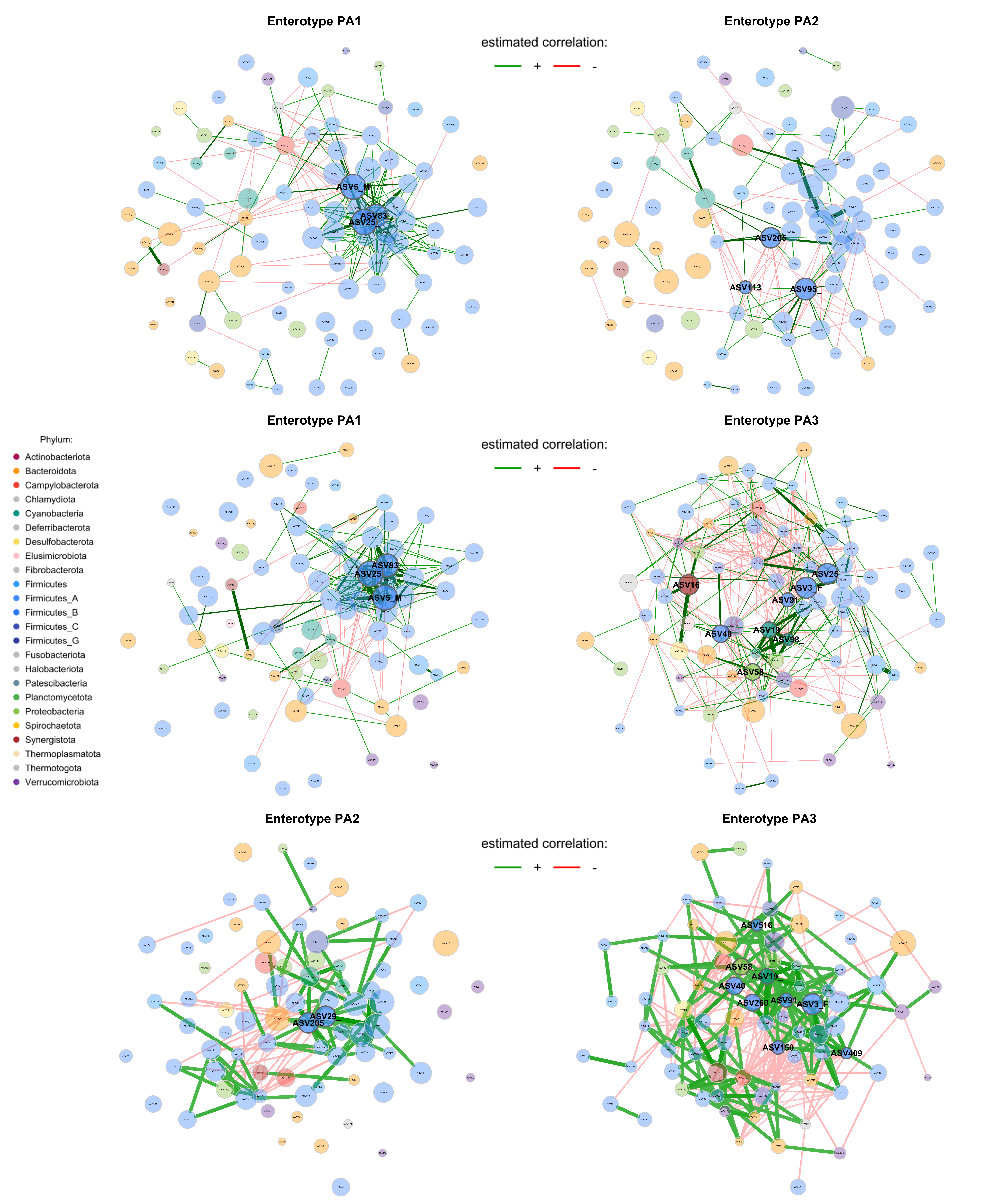

Supplement: Supplementary Data Sheet 1 — Table of geographical location and farming practices of the 60 farms. [file DataSheet_1.zip › Supplementary Data 11.PNG]

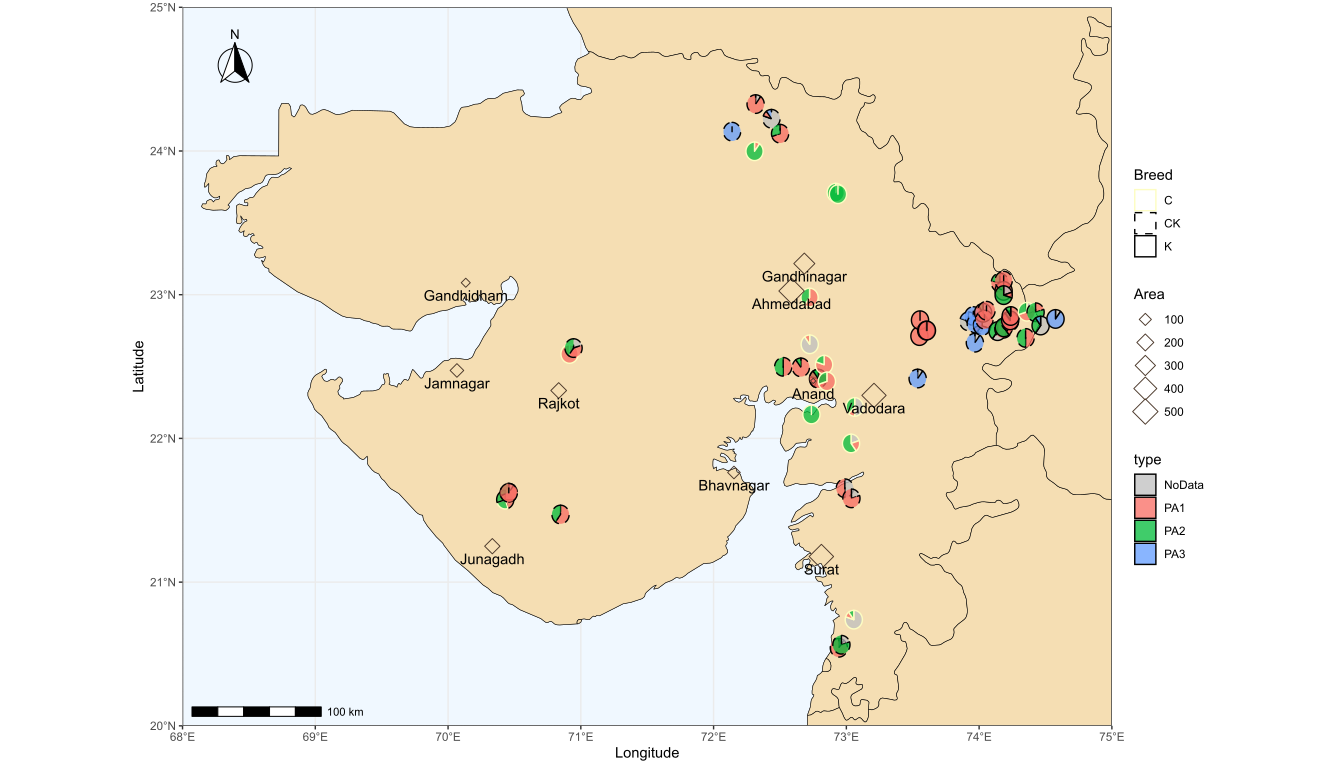

Supplement: Supplementary Data Sheet 1 — Table of geographical location and farming practices of the 60 farms. [file DataSheet_1.zip › Supplementary Data 14.PNG]

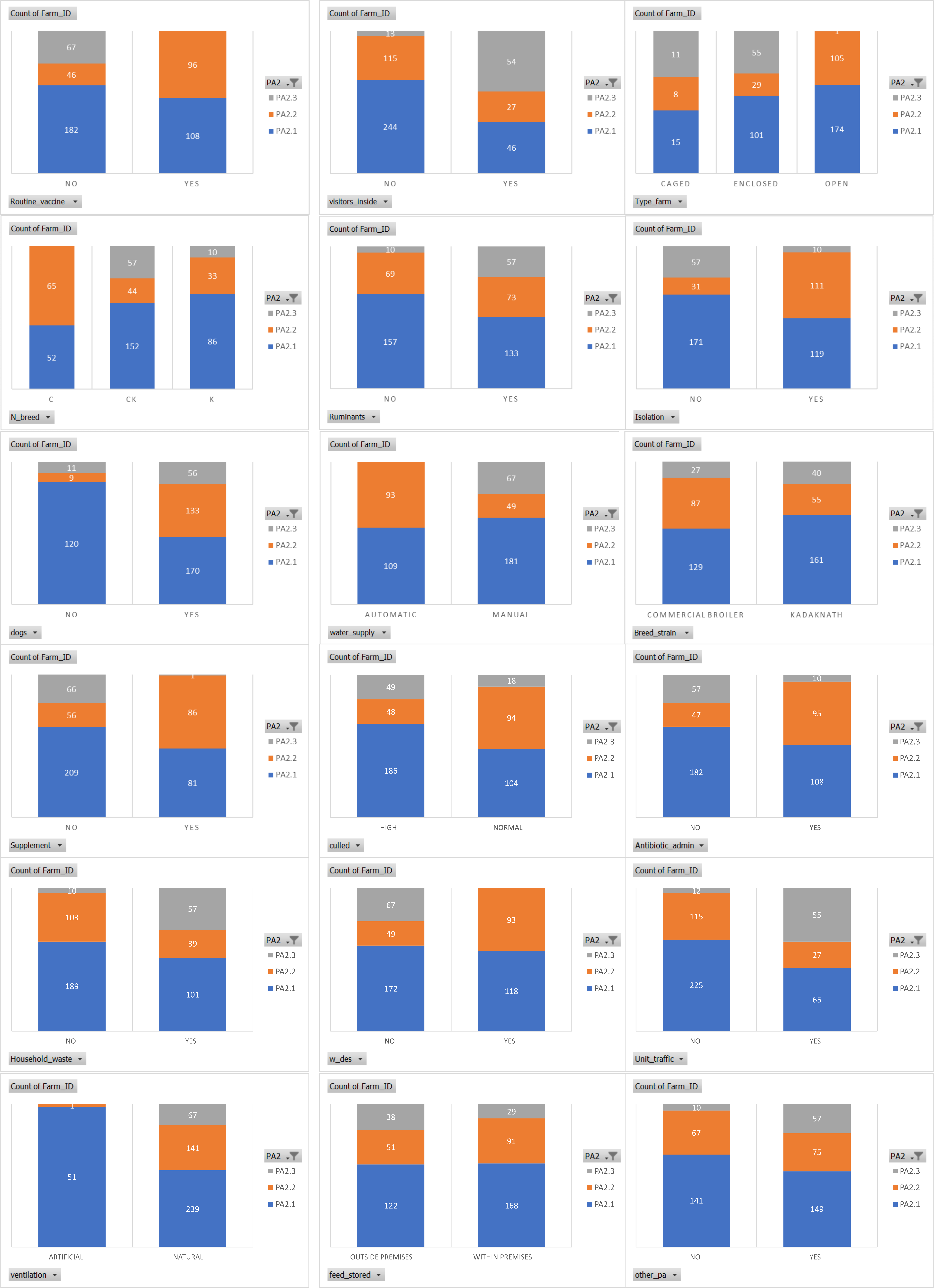

Supplement: Supplementary Data Sheet 1 — Table of geographical location and farming practices of the 60 farms. [file DataSheet_1.zip › Supplementary Data 15.png]
